# Not all screens are created equal: examination of surface features and other physical properties of commonly used screen materials for smoking drugs

**DOI:** 10.1186/s12954-023-00794-x

**Published:** 2023-05-23

**Authors:** Bradley J. Diak, Miroslav Miskovic, Nadia Zurba, Denise Beaumont

**Affiliations:** 1 Department of Mechanical and Materials Engineering, Queen’s University, Nicol Hall, 60 Union Street, Kingston, ON K7L-3N6 Canada; 2 Ontario Harm Reduction Distribution Program, Kingston Community Health Centres, 115 Barrack St - Suite 200, Kingston, ON K7K 1G2 Canada

**Keywords:** Crack cocaine, Brass screen, Steel wool, Stem filter, Materials characterization

## Abstract

**Background:**

Brass screens are considered an essential part of the safer drug smoking/inhalation supplies and are widely distributed by harm reduction programs in Canada. However, the use of commercially available steel wools as screens for smoking crack cocaine remains a common practice among people who smoke drugs in Canada. Use of these steel wool materials is associated with different adverse effects on health. This study aims to determine what changes folding and heating have on several filter materials, including brass screens and commercially available steel wool products, and examine the implications of these changes on health of people who smoke drugs.

**Methods:**

This study investigated the microscopic differences, studied by optical and scanning electron microscopy, between four screen and four steel wool filter materials used in a simulated drug consumption process. New materials were manipulated, compacted into its own Pyrex® straight stem using a push stick and then heated with a butane lighter simulating a common method in preparing drugs for consumption. The materials were studied in the as-received (new), as-pressed (compressed and inserted into the stem tube but without heating) and as-heated (compressed and inserted into the stem tube and heated with a butane lighter) conditions.

**Results:**

The steel wool materials with the smallest wire thicknesses were found to be the easiest to prepare for pipe use, but degrade significantly during shaping and heating, making them wholly unsuitable as a safe filter material. In contrast the brass and stainless steel screen materials remain mostly unchanged by the simulated drug consumption process. After the stainless steel pellet screen, the Brass Impact 2.0 screen material had the best characteristics of the materials tested due to its mesh wire diameter, pitch, alloy choice and its pre-strained state.

**Conclusion:**

Commonly used steel wool alternatives degrade during the handling and stem insertion, and heating the screens in the stem. Debris is generated by wool deformation on insertion and after heating that easily separates from the screen and can be inhaled during drug consumption. The brass and stainless steel screen materials are safer to use as they remain mostly stable during the simulated drug consumption process.

## Background

Brass screens are considered an essential part of the safer drug smoking/inhalation supplies. Brass screens are commonly included in safer smoking/inhalation kits and used in a conjunction with a straight stem. The Best Practice Recommendation for Canadian Harm Reduction Programs [1] recommend that all harm reduction programs should provide safer harm smoking/inhalation supplies, including brass screens to people who smoke drugs. A brass screen is moulded/shaped by hands and placed at one end of the straight stem to hold the solid crack cocaine in place and away from the mouth [1]. Push sticks are used to pack and position the filter or screen inside the crack pipe. When heating the Pyrex® stem, crack cocaine melts and releases vapours. The brass screens, originally developed by the tobacco industry for pipe smoking, have been repurposed as harm reduction supplies for safer drug smoking, permit the inhalation of drug vapour, while minimizing the inhalation of chemical residues, fragments, burning metal debris and smouldering crack particles that may cause foreign body trauma, burns and cuts to the lips, oral cavity, larynx and along the tract to the stomach and inflammation of the respiratory lining [2–9]. Cuts and sores could provide an entry point for bacteria and viruses. People who share crack pipes may be at increased risk of exposure to HCV and other communicable disease because HCV from drug blood residues on smoking paraphernalia (mouthpieces and stems) could pass into another person’s bloodstream through broken skin on their lips or mouth [10, 11].

Most common use of brass screens or steel wools for smoking drugs is as filters in straight stem Pyrex tubes to keep solid drugs, typically crack cocaine, in place while being heated, vapourized and inhaled. Other drugs, such as crystal meth, heroin and fentanyl, produces vapours when heated that could be inhaled but preparing these drugs for smoking typically involves using bowl pipes or foil without using brass screens or steel wools. Typical preparation method of crack cocaine for smoking begins with the preparation of straight stem. Several brass screens are twisted into a cone shape, inserted into the stem and pushed down the stem with a wooden stick. These tightly packed brass screens are positioned close to the opening of the straight stem on the side of the stem that is opposite to the side that goes into the person’s mouth. This method of packing brass screens is common but people who smoke drugs might use other methods.

A mouthpiece, which is a short piece of vinyl tube, is then placed on the end of the straight stem. It acts a barrier between the mouth and the straight stem preventing the heat from the stem to cause burns to the mouth. The solid crack rock is placed into a straight stem. The stem is heated by a flame, usually from a butane lighter that is placed underneath the pipe. This causes the crack rock to melt and release the vapour that is inhaled.

When access to brass screens is limited and/or because of individual preferences/habits for preparing crack cocaine, people who smoke drugs often use other potentially less-safe alternatives instead of brass screens to hold the drug when smoking. These alternatives typically involve commercially and widely available steel wool scouring pads from different manufacturers [5, 12–19]. A study by Leonard et al. [12] that examined HIV- and HCV-related risk practices among youth who smoke crack in Ottawa found that only a small proportion of both women (14%) and men (26%) reported that they ‘never’ or ‘rarely’ used a brass screen. However, the majority of both women (67%) and men (56%) in this group reported preference for steel wool or Brillo® as the main reason for not using brass screens.

Frequently people who smoke drugs refer to these steel wool alternatives to brass screen by the brand name ‘Brillo®’, which is a specific brand of scouring pads but is also commonly used as an umbrella term for all steel wool pads. Steel wool pads, being relatively inexpensive and widely available at local convenience stores and supermarkets, are easy to obtain which contributes to the widespread use of these products among people who smoke drugs [20].

Despite the wide distribution of brass screens in harm reduction programs in Canada, reports and studies reported persistent use of steel wool products [21–25]. A survey of individuals who smoked crack in Vancouver’s inner city found that only 42% of kit recipients reported using brass screens and 91% reported usually or always using Brillo®, despite that brass screens were included in the safer smoking/inhalation kits [24]. In 2006, a Safer Crack Outreach, Research, and Education (SCORE) survey of 126 women and 80 men in Vancouver’s Downtown Eastside (DTES) conducted prior to kit construction and distribution suggested a high incidence of Brillo® use (98.4%) [19]. In 2015, 812 clients completed a survey at 34 harm reduction sites across five health authorities in BC. The survey found that of all people who reported smoking crack cocaine, 33% reported using a brass screen while 78% used Brillo® [26].

A study by Boyd et al. [27] found that the most commonly reported factors associated with the preference for Brillo® over brass screens among people who use drugs were: easier handling when in a rush, shorter time to insert Brillo® in the stem and long-lasting habit of using Brillo®. The same study found that the changes in drug smoking practices are less likely to occur if harm reduction equipment requires more time to use, is awkward to use, hinders consumption or leads to loss of the drug [27]. Hopkins et al. [21] attributed the continued use of metal wools such Brillo® to its ease of use. The research highlights the importance of repeated messaging about safer crack use from peers and outreach workers and providing education to clients about advantages and preparation methods for brass screens in order to shift personal crack use practices [24, 27]. In order to maximize the use of safer smoking/inhalation supplies, the supplies should be designed to meet the needs of people who smoke drugs. Understanding and documenting the difficulties that people who smoke drugs experience when handling and using the supplies could inform the design of harm reduction supplies to maximize their adoptability and use [28].

Steel wool products are not designed to be used as screens for smoking drugs, and they are more likely to disintegrate faster when being handled and inserted into the stem and heated then brass screens, the latter which are distributed by harm reduction programs. When smoking drugs, these steel wool products may break apart into fragments which are then inhaled and can cause injuries to the oral cavity, larynx and lungs [7, 14–18, 29–34]. Several studies reported that negative health consequences associated with using Brillo® for smoking drugs are common among people who use drugs and include: inhaling the whole Brillo® screen, developing burns and cuts on lips, developing cuts on fingers when handling Brillo® and breathing difficulties [17, 20, 24, 27, 33, 35]. In a study about structural inequities influencing the health of street-involved women who use illegal drugs in Vancouver, 51 (41.1%) women who smoked crack cocaine of total 126 participants reported inhaling Brillo® in mouth, throat or lungs in the past year [20]. The black sputum (phlegm), that was reported by 75% of participants in a study of respiratory issues among people who smoke crack cocaine in Toronto, might be caused by the inhaled burnt steel wool fragments [36].

Brass screens distributed by harm reduction programs are less likely to break apart than steel wool or Brillo® and are not coated with potentially toxic substance [24]. Some commercial steel wool products are coated with substances, such as soap and cleaning products that could be inhaled when the product is heated (e.g. Brillo® and Chore Boy®) [1]. Additional harms associated with steel wool use include inhaling toxic volatile organic carbons released when steel wool is burned [37]. Often, people who smoke drugs will heat the steel wool with a lighter to burn off the coating before using it as a screen for the first time. If a client is unwilling to use brass screens rather than steel wool, some harm reduction programs encourage their clients to place brass screens between the steel wool and the mouth or wrap steel wool in brass screens to act as a barrier for loose shards [37].

The Ontario Harm Reduction Distribution Program (OHRDP), a program of Kingston Community Health Centres, is non-profit organization. OHRDP is a provincial program which coordinates the distribution of evidence based harm reduction supplies to Core Harm Reduction Programs throughout Ontario, which then distribute supplies to community agencies and mobile services. Total number of access points giving out free harm reductions supplies at the end of 2021 was 466. OHRDP has been distributing Brass Impact 1.0 screens in Ontario since July 2019. Brass screens provided through OHRDP are safer for use as screen for smoking drugs than other commercially available steel wool products. Brass screens are made from a weave of small diameter wires. The brass screens are high heat resistant, malleable, and have no chemical coating. Brass screens are packaged in a packet containing five brass screens (OHRDP, personal communication). Use of alternative materials like steel wools for screens is likely less safe than using brass screens due to the risk of inhaling the hot screen material [37, 38].

Inhalation of crack cocaine vapours, which is commonly referred to as ‘smoking crack cocaine’, differs from the traditional notion of smoking that is used in the context of tobacco and marijuana cigarettes. When a cigarette is lit, the tobacco or cannabis inside it undergoes a process of combustion, which releases heat and energy, and produces smoke and ash. The combustion process is self-sustaining because the heat and energy released by the burning tobacco provide enough energy to keep the process going, as long as there is enough tobacco and oxygen available.

Crack is the freebase form of cocaine that has a lower melting point (96–98 °C) than cocaine hydrochloride (198 °C), resistance to thermal degradation and lipid solubility. When freebase cocaine is heated, it quickly melts and releases particulate matter and vapour, which are inhaled and absorbed by the lungs. Therefore, ‘smoking’ crack cocaine, crystal meth or opioids does not involve inhaling smoke produced by direct ignition. Instead, in the case of crack cocaine, it involves heating a Pyrex stem containing the substance enclosed in a screen to generate an aerosol through the condensation of a vapour. This non-combustible mechanism of delivery of crack cocaine is similar to hookah smoking (specially prepared molasses-based tobacco product is placed on charcoal and covered with aluminium foil with holes in it), e-cigarette vaping and heated tobacco products.

This study examines the effects of folding and heating on several types of brass screens that are distributed by harm reduction programs and potentially unsafe but commonly used screen alternatives like steel wool products. This study examined the behaviour of brass screens and steel wools when exposed to heat in the simulated process of smoking crack cocaine. The experiments were performed in the absence of drug because the focus was on how the behaviour of the equipment used for smoking crack cocaine during the drug preparation process (manipulation and heating of brass screens and steel wools) has implications on safer drug smoking practices. To our knowledge, no study has sought to characterize filter materials commonly used for smoking drugs and effects that folding and heating in a straight stem pipe have on these materials. The over-arching objective of this study is to identify what filter material characteristics might give people who smoke drugs the best and safest experience.

## Materials and methods

### Study methodology

The methodology used optical and scanning electron microscopy (SEM) to characterize the microscopic differences of four screen and four steel wool filter materials used in a drug consumption process**.** The materials were examined before and after laboratory manipulation and heating in a process that simulated the techniques for the preparation of drugs for smoking/inhalation used by people who smoke drugs. The materials were examined in the as-received, as-pressed and as-heated conditions in order to evaluate the drug consumption methodology’s effect on the materials without using drugs.

### Materials

Eight filter materials and related supplies were procured by OHRDP for the investigation. The materials included four screen and four wool materials, new Pyrex® stems, birch push sticks and BIC® *Mini* butane lighters. The complete list of filter materials received for study is shown in Table 1 and is shown in their original packaging in Appendix A. All as-received filter materials were brand new, unused and clearly labelled, but without information about the original manufacturers.

**Table 1:** List of filter materials examined by optical and scanning electron microscopy

SCREENS	Brass impact 1.0	Brass black packet	Brass Impact 2.0	Terpan Prévention KitBase® Stainless Steel Pellet Screen
WOOLS	Scrubber CleanZ	Bull Dog Medium Steel Wool	Rhodes American Steel Wool	S.O.S Steel Wool Pads

### Experimental conditions

The materials were studied in the following conditions: 1) as-received (in their unused and from the original packaging condition); 2) as-pressed (manipulated by hand and positioned in the stem using a push stick; the materials were positioned in the stem using a simulated screen preparation and positioning process and examined before heating the stem); and 3) as-heated (after heating the materials in the straight stem with a butane lighter during a simulated drug consumption process). All materials in all three experimental conditions were examined without drugs present.

### Condition 1: ‘As-received’

All as-received filter materials were ultrasonically cleaned in an ethanol bath for two minutes and hot air-dried before use, unless otherwise stated. This condition will also be referred to as neat. Single fibres of wool were specifically separated from the bulk clump for easier as-received characterization. All steel wools were first heated ex situ using the BIC® *Mini* butane lighter in the neat state to burn off possible residue on the materials before compaction and heating in the tube (in situ).

### Condition 2: ‘As-pressed’

This process simulates the condition of the filter material being prepared in the stem before the drug is heated. Following the instructions from shared online material and booklet literature provided and created by OHRDP (Strike et al. [1]) on one method for manipulating and inserting screens in the stem, each material was manipulated and compacted into its own Pyrex® straight stem using a push stick. For screens, the compressed wads consisted of four screens either pre-stacked or compressed serially one by one. The stainless steel pellet screen was compacted into its own Pyrex® tube which was part of the Terpan Prévention KitBase®.

### Condition 3: ‘As-heated’

This process step simulates the condition of the material after heating the stem using a butane lighter simulating a drug heating process. The compacted filter, or wad, was heated in situ in the Pyrex® tube for 20 s using a BIC® *Mini* butane lighter.

### Materials characterization

Materials characterization consisted of imaging use both optical microscopy (OM) and scanning electron microscopy (SEM) at Queen’s University Department of Mechanical and Materials Engineering. An Olympus SZX7 stereo-microscope was used for OM. An FEI Nova NanoSEM 450™ with Bruker XFlash 6160 detector was operated at 20 kV for high magnification imaging and qualitative chemical analysis by energy-dispersive spectroscopy (EDS). All specimens were ultrasonically cleaned in ethanol for two minutes and hot air-dried before imaging in the SEM to enhance imaging conditions and minimize contamination in the microscope. All SEM images reported used secondary electron imaging (SEI) mode.

## Results

### ‘As-received’ condition

The characterization of the as-received materials was done to quantify the size (wire cross section) and shape of the fibres that make the mesh, confirm its chemical composition (i.e. brass, steel, stainless steel) and generally observe the cleanliness of the materials’ surfaces. Mesh geometries can be characterized by three parameters (Appendix B): wire diameter (d), pitch (p) and aperture width (w). The results are presented for screens first followed by wools.

**Fig. 1:**
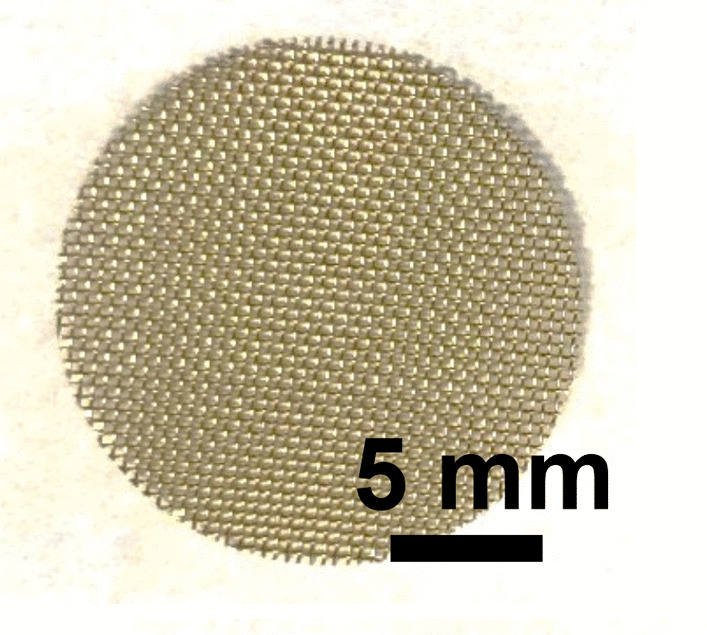
OM image of *as-received* Impact 1.0 brass screen

**Fig. 2:**
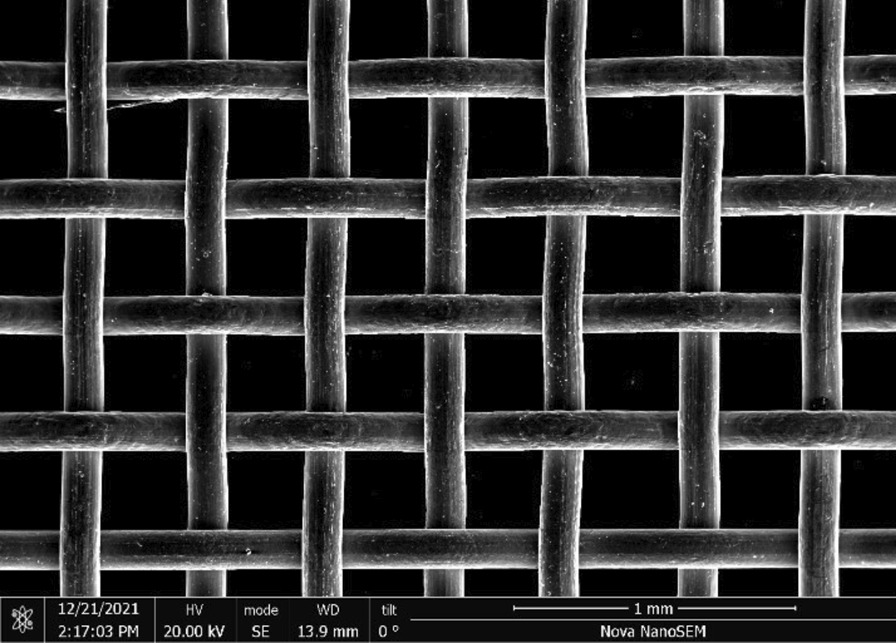
SEM-SEI of *as-received* Impact 1.0 square weave. Scale bar is 1 mm

**Fig. 3:**
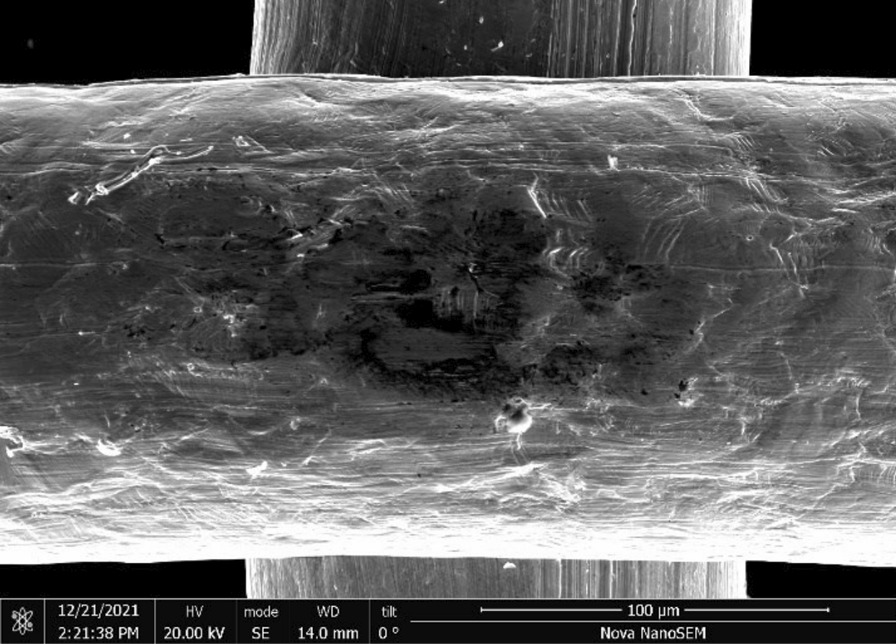
SEM-SEI of *as-received* Impact 1.0 wire surface. Scale bar is 100 µm

**Fig. 4:**
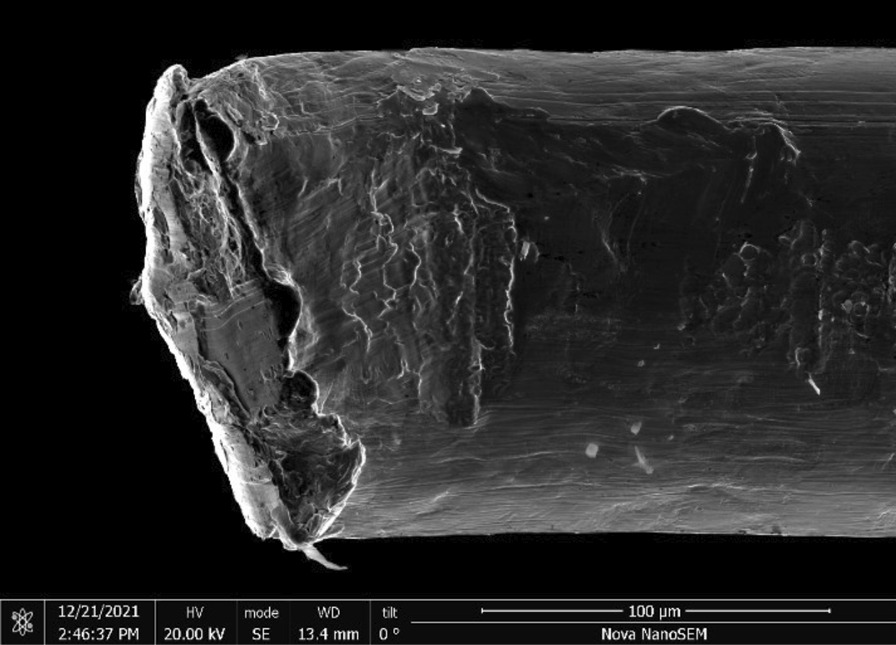
SEM-SEI of *as-received* Impact 1.0 wire end. Scale bar is 100 µm

Brass Impact 1.0—Brass Impact 1.0 is the screen material supplied most recently in Ontario for harm reduction (Fig. 1). The screen is a 58 × 58 mesh (Fig. 2) consisting of 145 μm diameter brass wires in a plain weave with 0.5 mm pitch and 0.28 mm aperture width (Fig. 3). As a comparison, the average human hair diameter is about 100 μm. The chemical composition of the wire is predominantly copper and zinc in the compositional range of an *α* + *β* brass alloy with a melting temperature of about 903 °C. The surfaces of the wire have axial grooves due to the drawing process and are overall clean (Fig. 4). The ends of the wires are jagged likely due to shearing to obtain the overall circular shape of the screen.

**Fig. 5:**
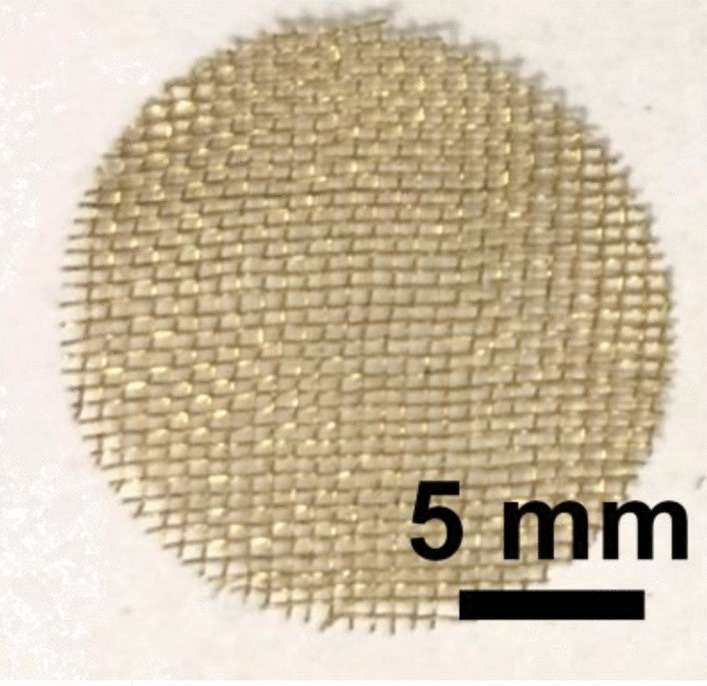
OM image of *as-received* Brass Black Packet

**Fig. 6:**
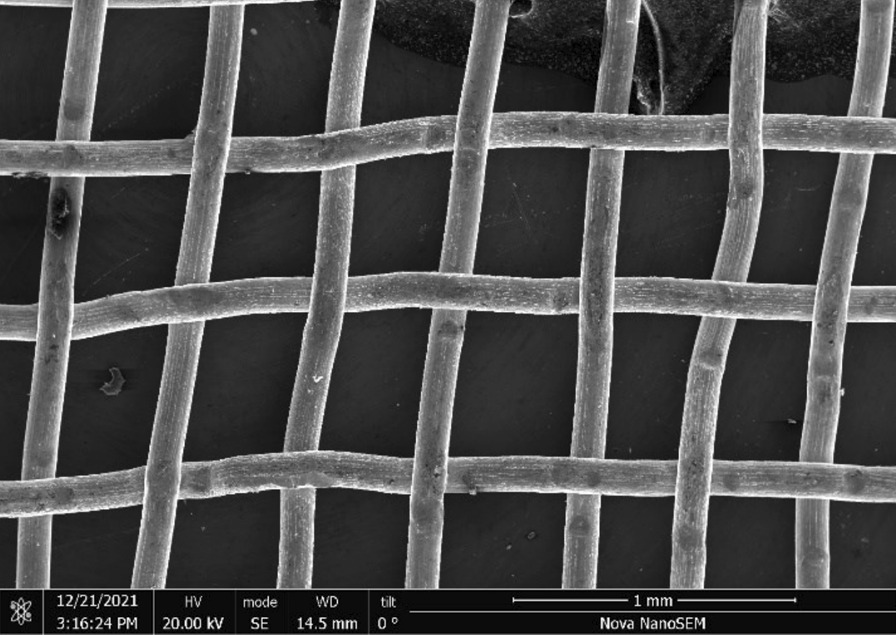
SEM-SEI of *as-received* Brass Black Packet non-uniform weave. Scale bar is 1 mm

**Fig. 7:**
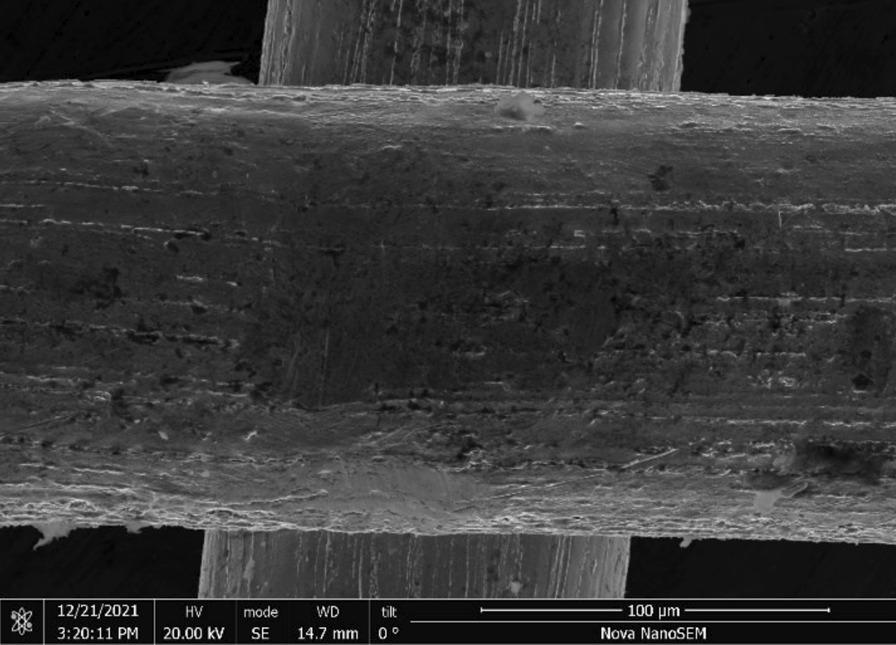
SEM-SEI of *as-received* Brass Black Packet wire surface. Scale bar is 100 µm

**Fig. 8:**
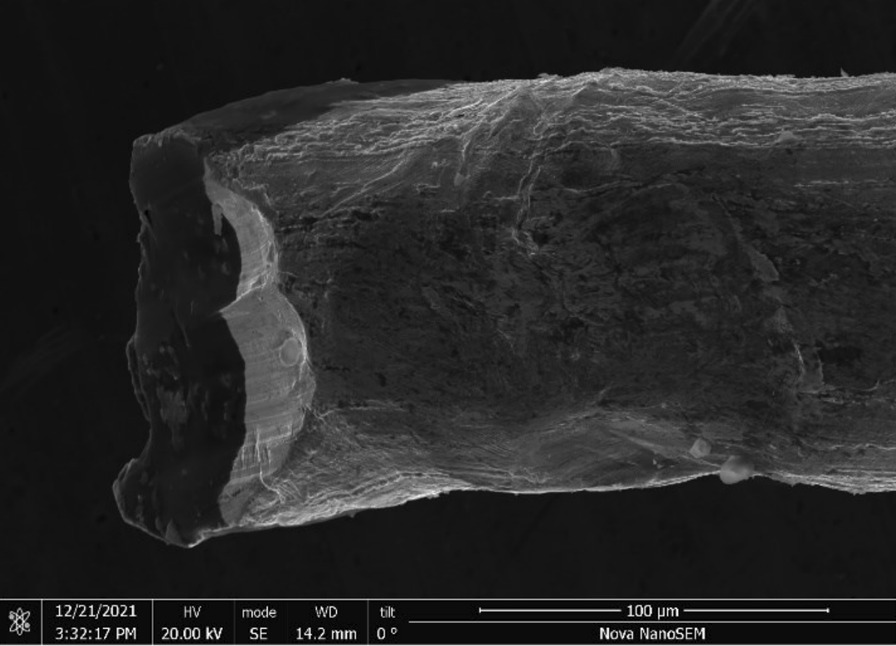
SEM-SEI of *as-received* Brass Black Packet wire end. Scale bar is 100 µm

Brass Black Packet (made in India)—The Brass Black Packet (Fig. 5) is a 55 × 55 mesh (Fig. 6) consisting of 130 μm diameter brass wires a plain weave with non-uniform 0.67 mm pitch and non-uniform 0.36 mm aperture width (Fig. 7). The smaller diameter wire and larger pitch lead to looser weave with more separation at the ends. The wire surfaces show drawing lines similar to Impact 1.0, but with more abrasion roughness. The ends of the wires also appear sheared (Fig. 8) to obtain the overall circular screen geometry.

**Fig. 9:**
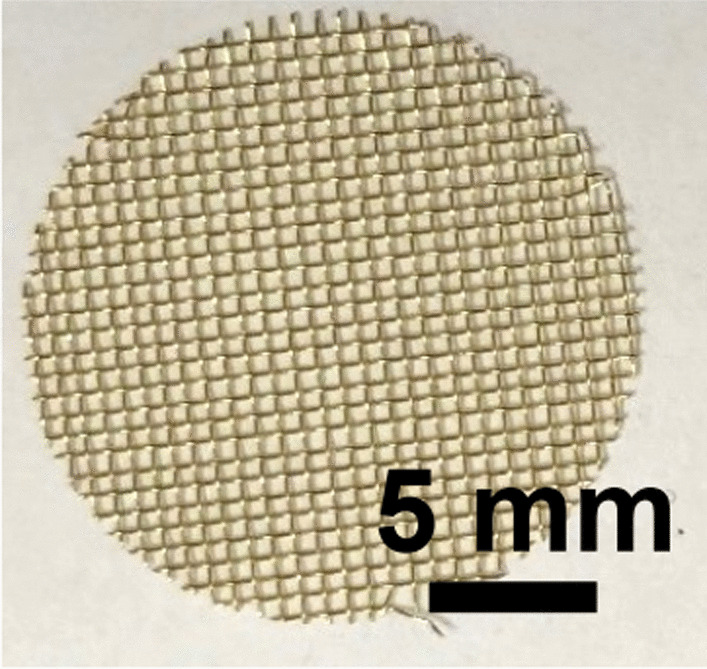
OM image of *as-received* Impact 2.0 brass screen

**Fig. 10:**
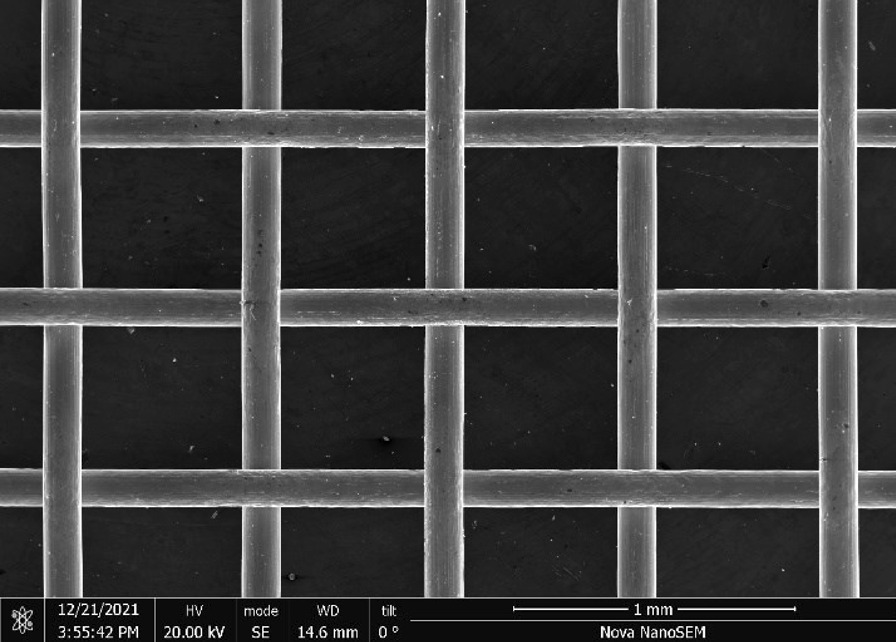
SEM-SEI of *as-received* Impact 2.0 square weave. Scale bar is 1 mm

**Fig. 11:**
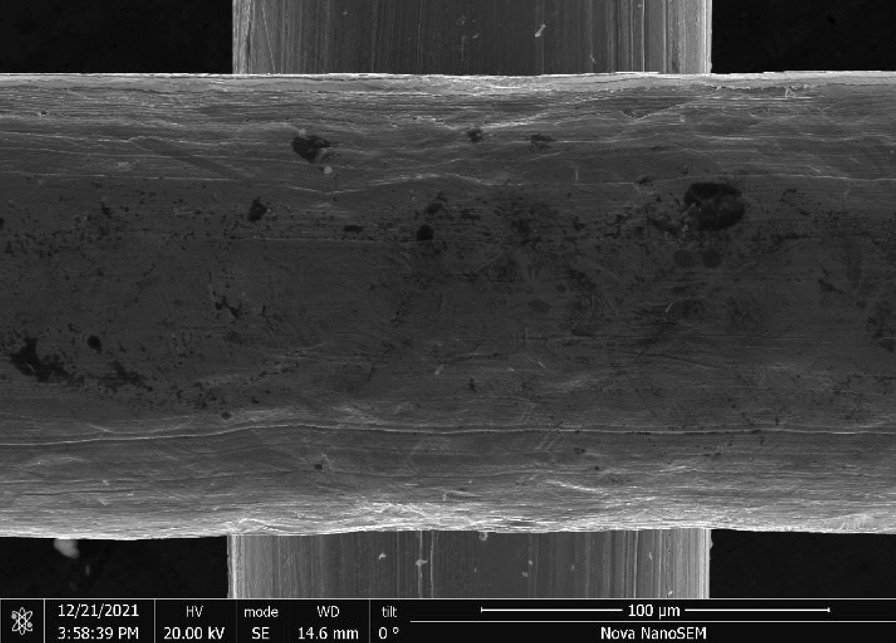
SEM-SEI of *as-received* Impact 2.0 wire surface. Scale bar is 100 µm

**Fig. 12:**
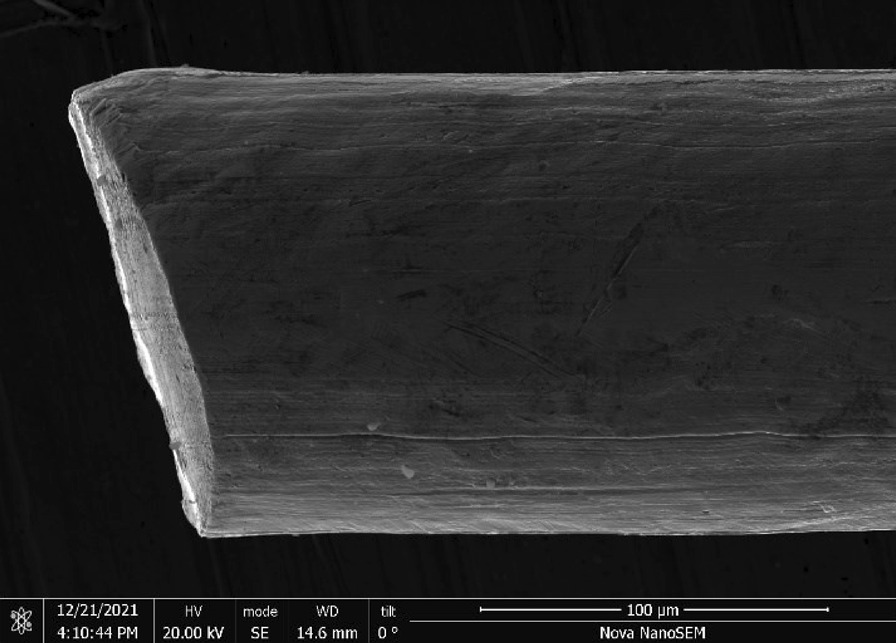
SEM-SEI of *as-received* Impact 2.0 wire end. Scale bar is 100 µm

Brass Impact 2.0—Brass Impact 2.0 is also available through OHRDP to Ontario harm reduction programs. The screen aperture is more open than the Impact 1.0 with a 37 × 37 mesh (Fig. 9) consisting of 137 μm diameter brass wires in a plain weave with 0.92 mm pitch and 0.52 mm aperture width (Fig. 10). The surface roughness (Fig. 11) and composition are similar to Impact 1.0. The wire ends are also sheared (Fig. 12) likely having been punched out of a larger screen sheet to obtain the generally circular screen geometry.

**Fig. 13:**
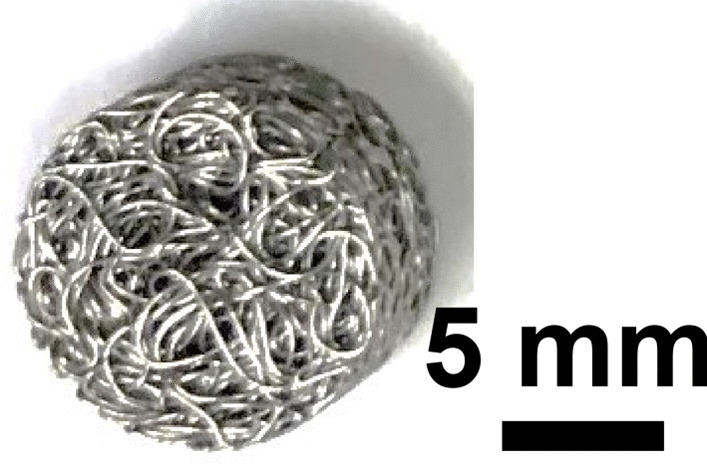
OM image of *as-received* stainless steel pellet screen

**Fig. 14:**
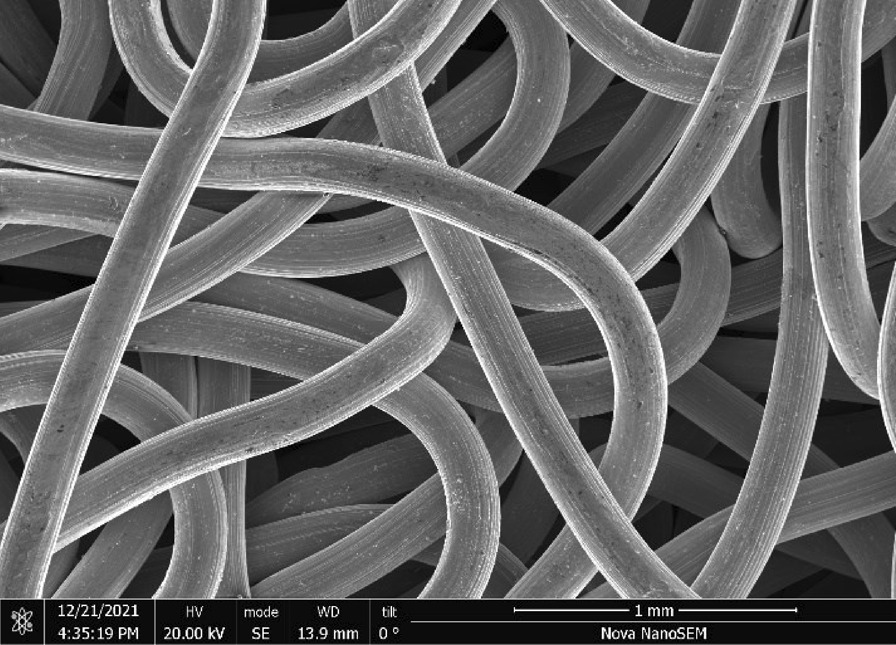
SEM-SEI of *as-received* pellet screen surface. Scale bar is 1 mm

**Fig. 15:**
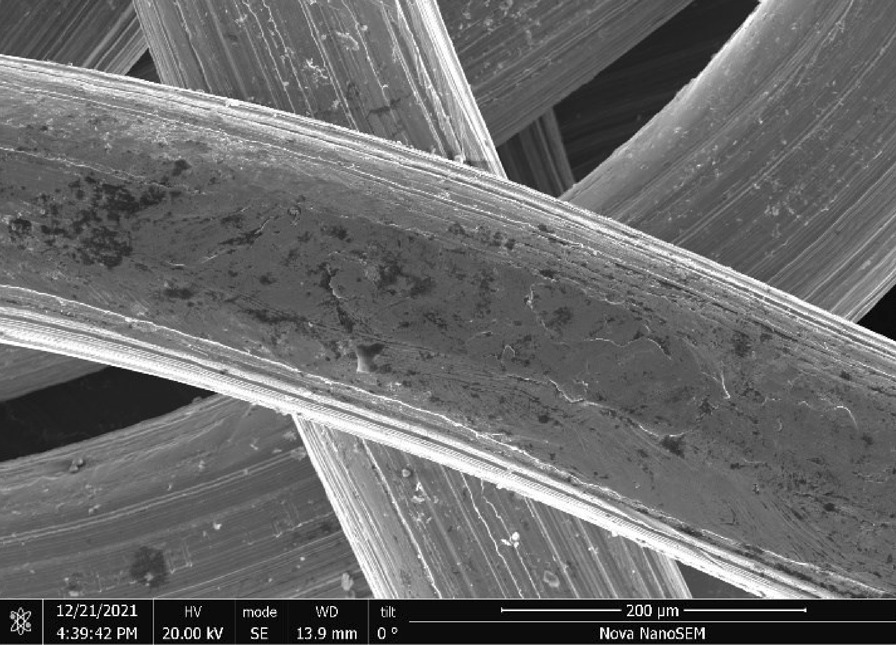
SEM-SEI of *as-received* pellet screen surface. Scale bar is 200 µm

**Fig. 16:**
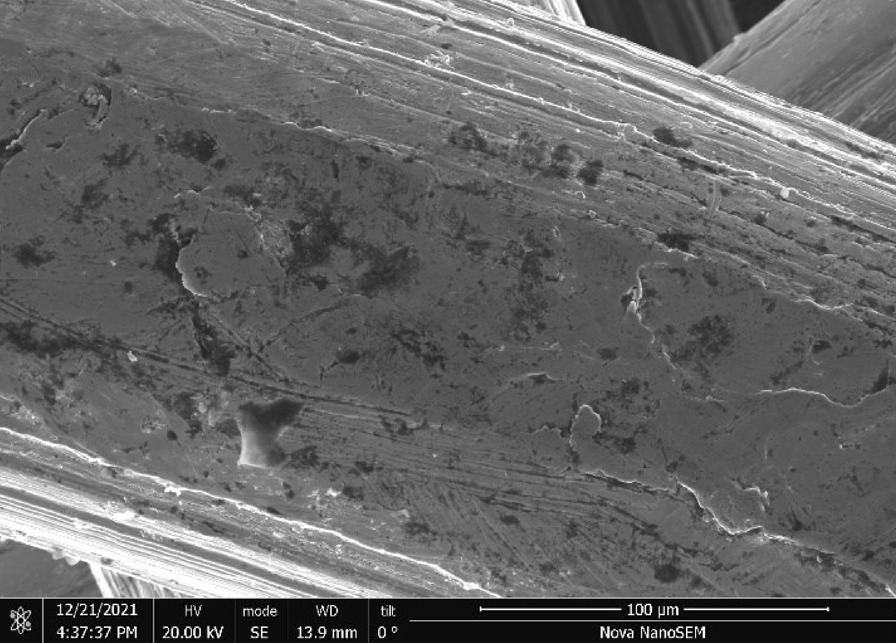
SEM-SEI of as-received pellet screen surface. Scale bar is 100 µm

Stainless Steel Pellet Screen—The stainless steel pellet screen is a wire compressed into an overall cylindrical shape (Fig. 13). No free-ends were observed suggesting that the screen is made from one single continuous wire (Fig. 14). The wire has surface markings typical of wire drawing (Fig. 15) and has a diameter of 185 μm (Fig. 16). The wire predominantly contains iron, chromium and nickel typical of a ferritic stainless steel.

**Fig. 17:**
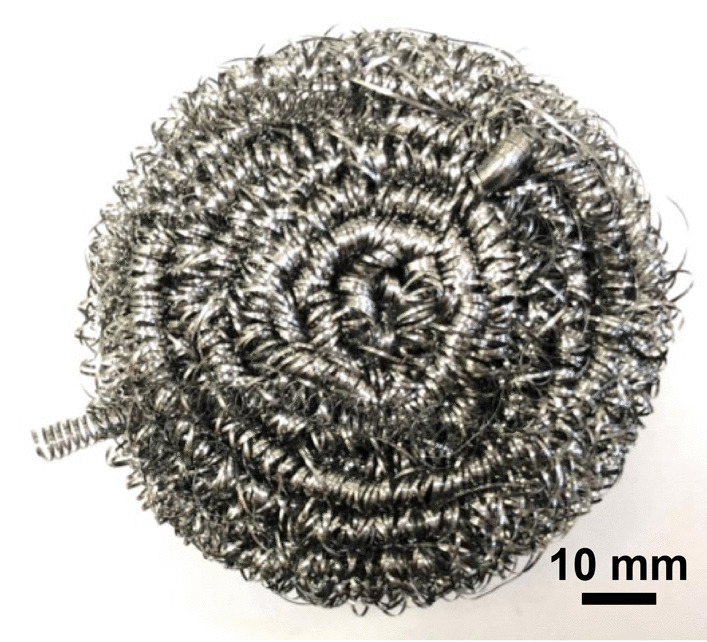
OM image of *as-received* Scrubber CleanZ pad

**Fig. 18:**
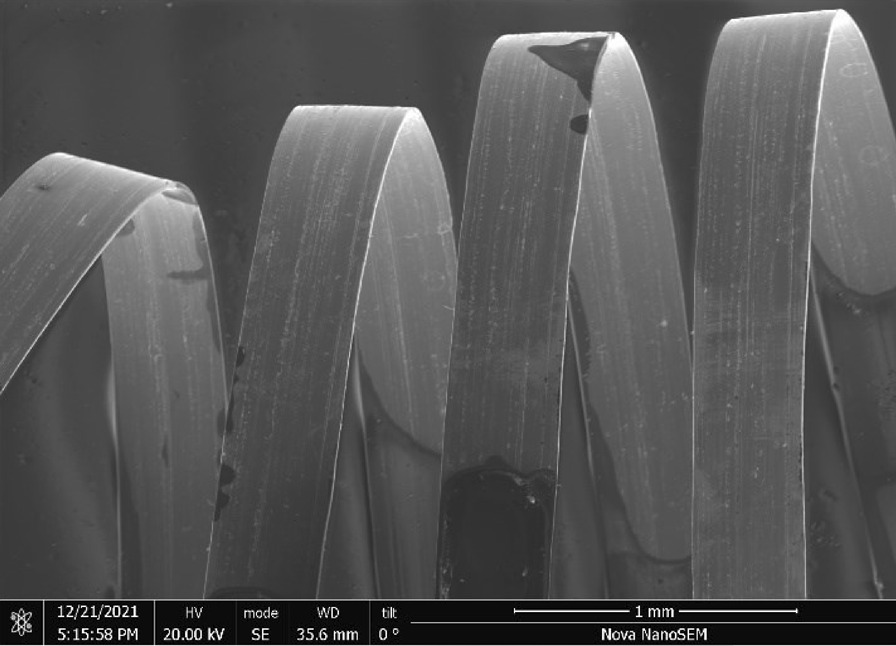
SEM-SEI image of *as-received* Scrubber CleanZ ribbon. Scale bar is 1 mm

**Fig. 19:**
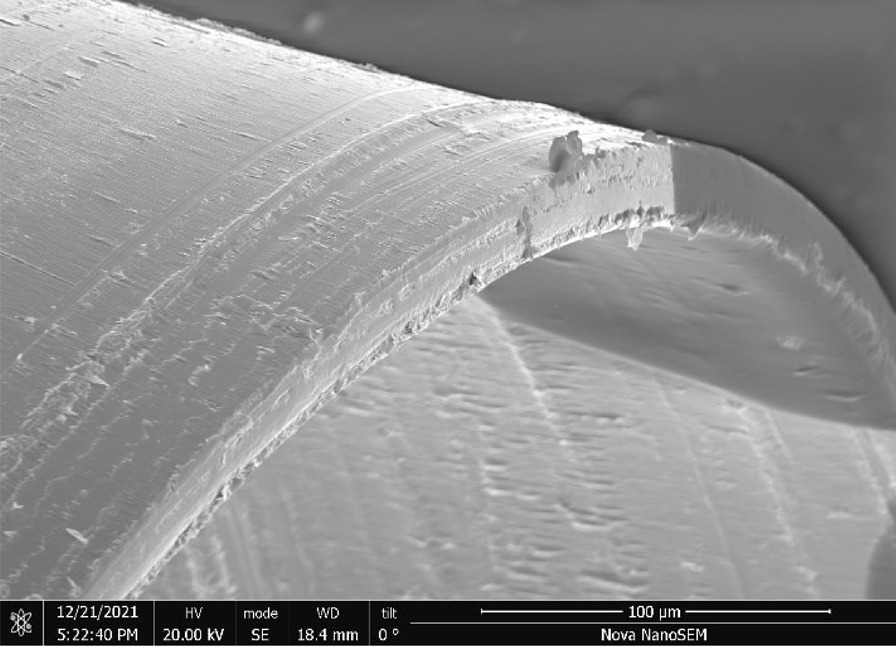
SEM-SEI image of *as-received* Scrubber CleanZ ribbon surface. Scale bar is 100 µm

**Fig. 20:**
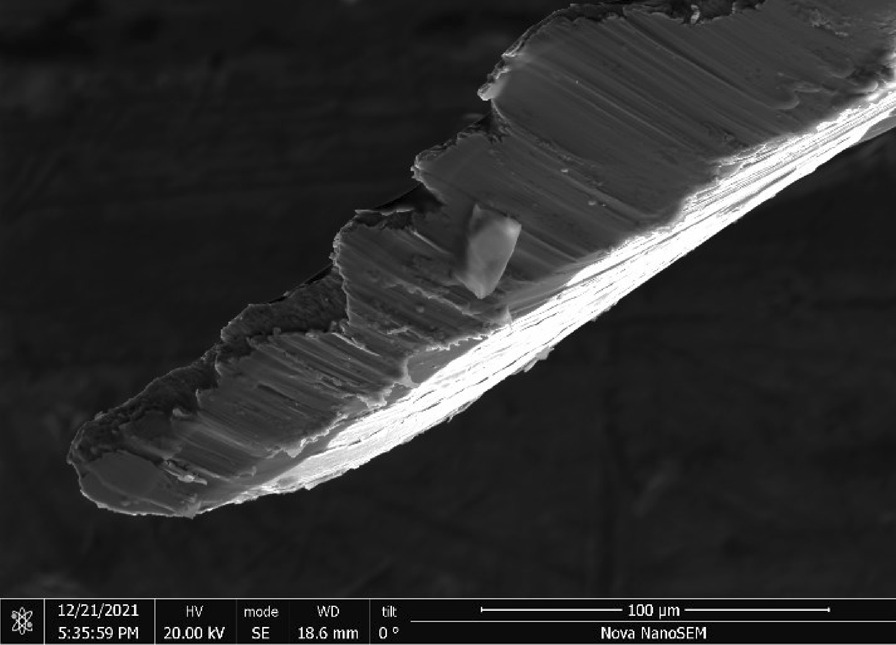
SEM-SEI image of *as-received* Scrubber CleanZ ribbon end. Scale bar is 100 µm

Scrubber CleanZ—The CleanZ scrubber (Fig. 17) wires have a coiled ribbon-like geometry (Fig. 18) that are 0.4 mm wide and 21 μm thick (Fig. 19). The main chemical constituents are iron, chromium and nickel typical of a ferritic stainless steel. The ribbon sides and ends have sharp edges (Fig. 20).

**Fig. 21:**
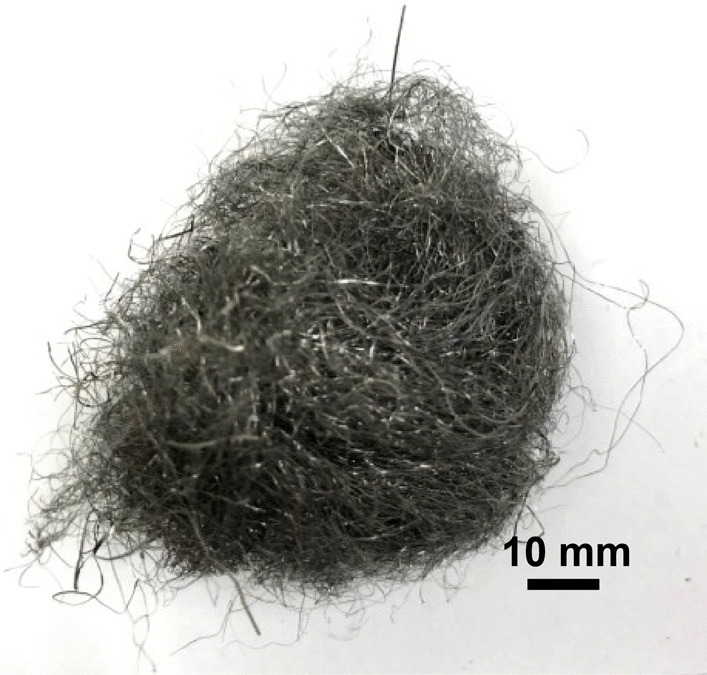
OM image of *as-received* Bull Dog steel wool

**Fig. 22:**
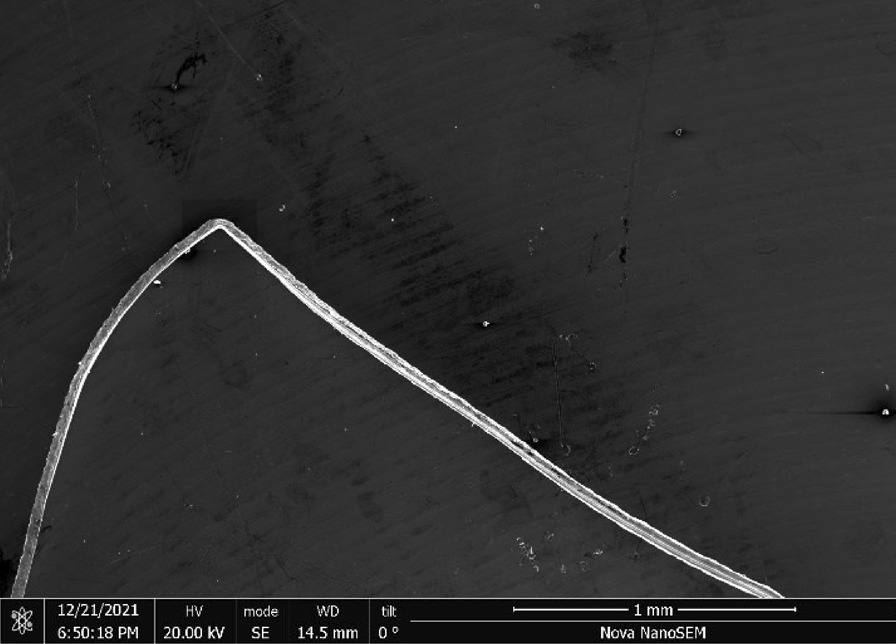
SEM-SEI image of *as-received* Bull Dog wire with kink. Scale bar is 1 mm

**Fig. 23:**
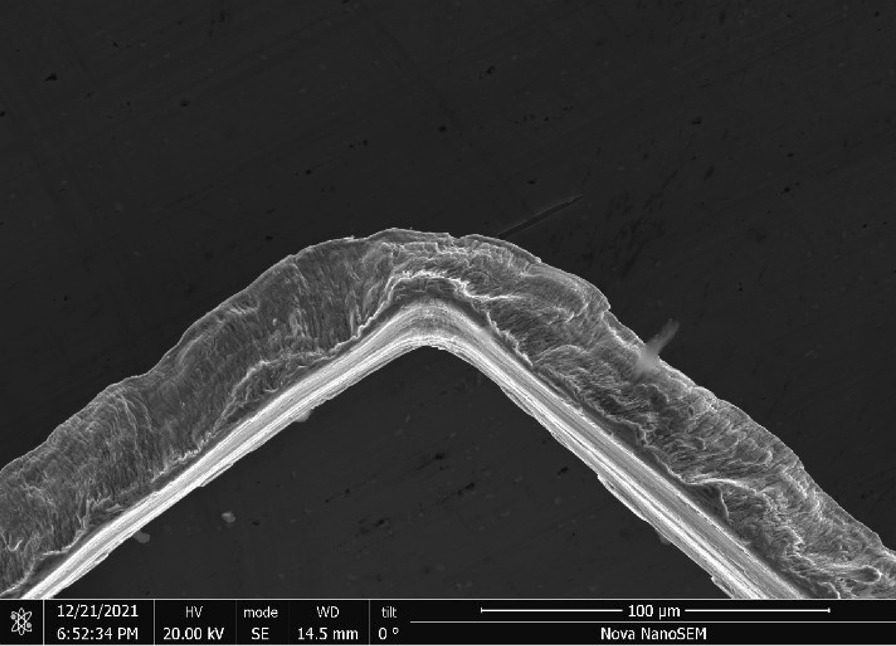
SEM-SEI *as-received* Bull Dog wire with kink showing the surface. Scale bar is 100 µm

**Fig. 24:**
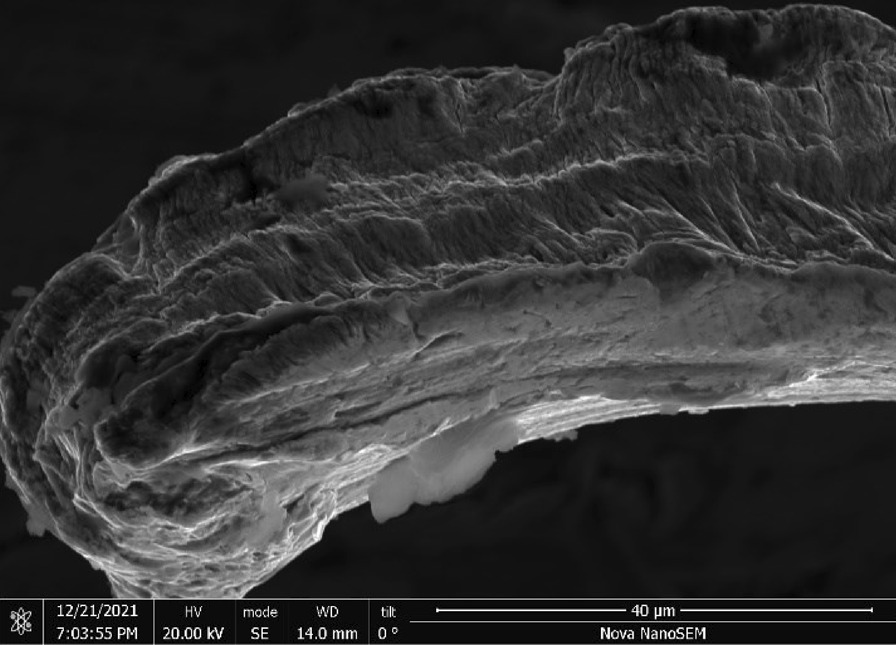
SEM-SEI image of *as-received* Bull Dog wire end. Scale bar is 100 µm

Bull Dog—Bull Dog medium steel wool (Fig. 21) consists of wire strands, often kinked (Fig. 22), with a range of cross sections from 1 mm × 0.05 mm down to 0.05 mm × 0.05 mm (Fig. 23). The composition is primarily iron and resembles a carbon steel. Note that carbon was undetectable by the measurement technique used. The strand ends were rough (Fig. 24).

**Fig. 25:**
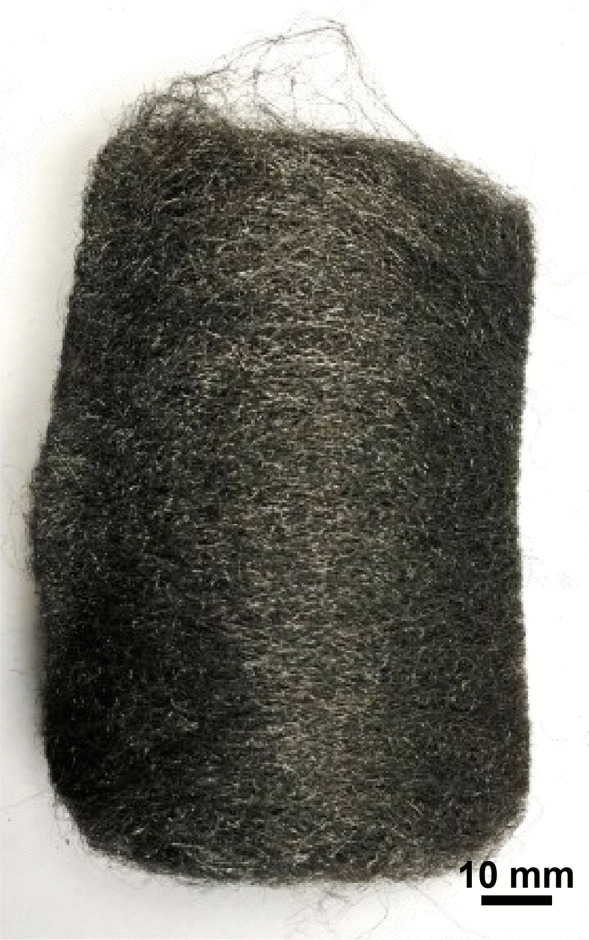
OM image of *as-received* Rhodes American steel wool

**Fig. 26:**
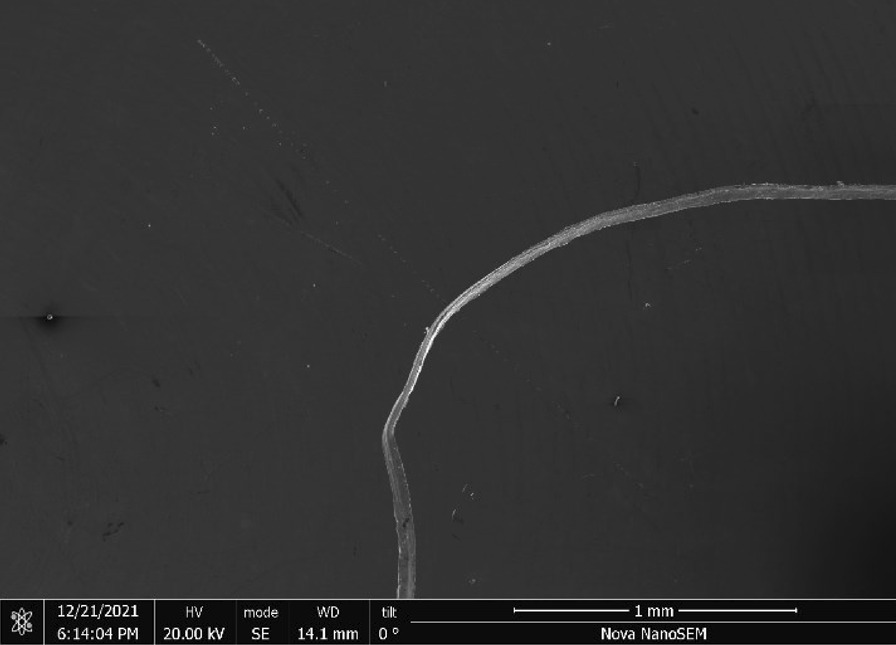
SEM-SEI image of *as-received* Rhodes American wire. Scale bar is 1 mm

**Fig. 27:**
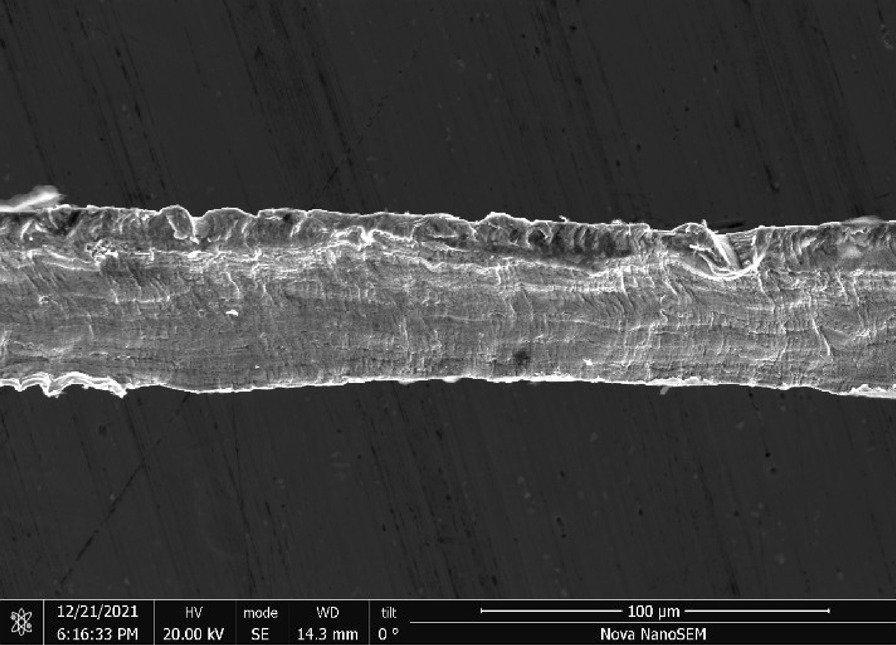
SEM-SEI image of *as-received* Rhodes American wire surface. Scale bar is 100 µm

**Fig. 28:**
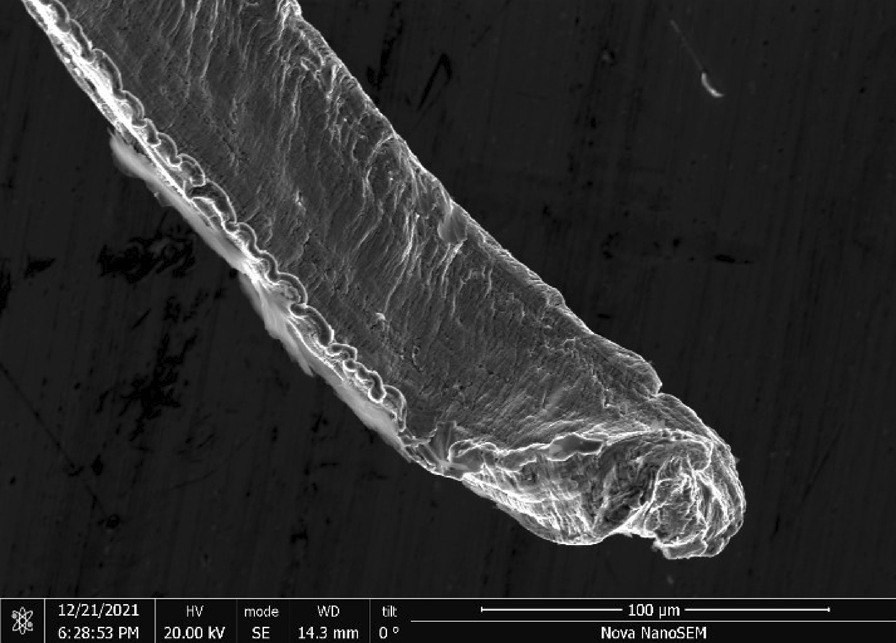
SEM-SEI image of *as-received* Rhodes American wire end. Scale bar is 100 µm

Rhodes American Steel Wool—Rhodes American steel wool grade #00 (Fig. 25) consists of wire strands (Fig. 26) with consistent 0.05 mm × 0.05 mm cross section (Fig. 27). The chemical composition resembles a typical carbon steel. The manufacturing process leads to rough surfaces and wires with deformed ends (Fig. 28)

**Fig. 29:**
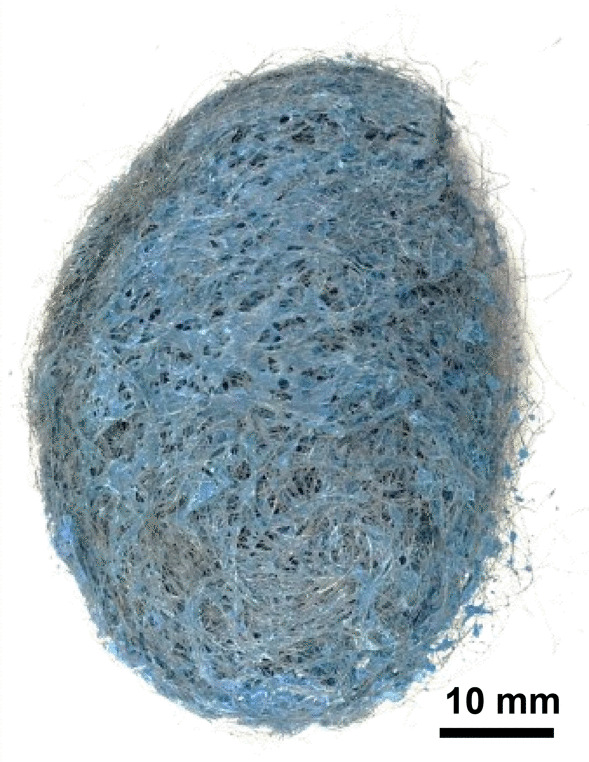
OM image of *as-received* S.O.S. steel wool pad

**Fig. 30:**
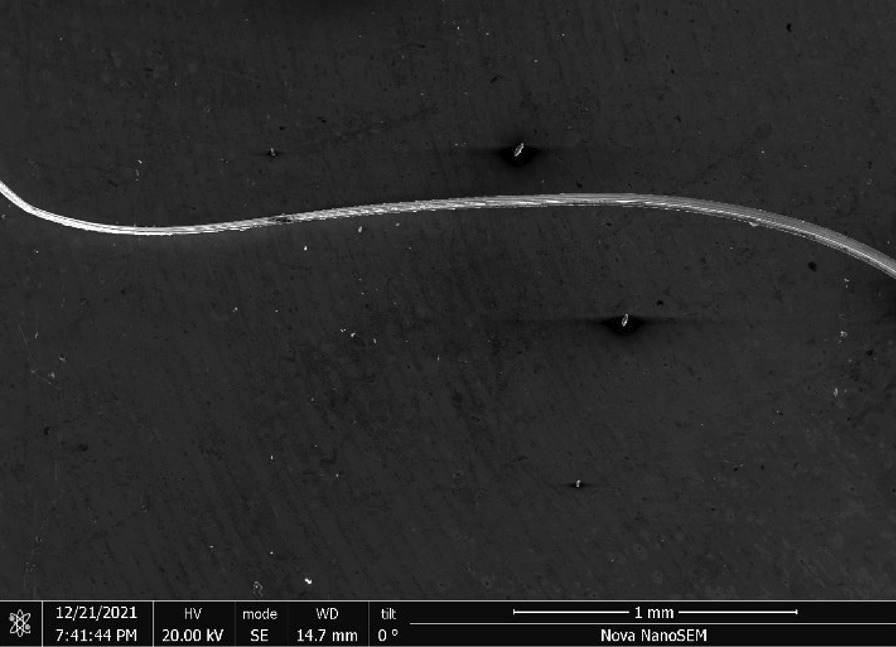
SEM-SEI image of *as-received* S.O.S. wire. Scale bar is 1 mm

**Fig. 31:**
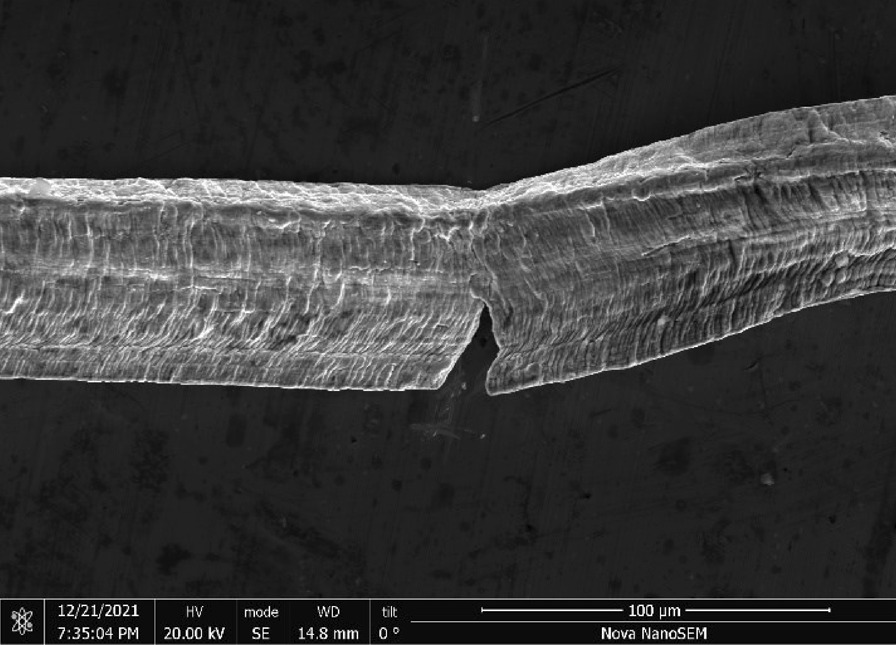
SEM-SEI image of *as-received* S.O.S. wire surface showing inherent crack. Scale bar is 100 µm

**Fig. 32:**
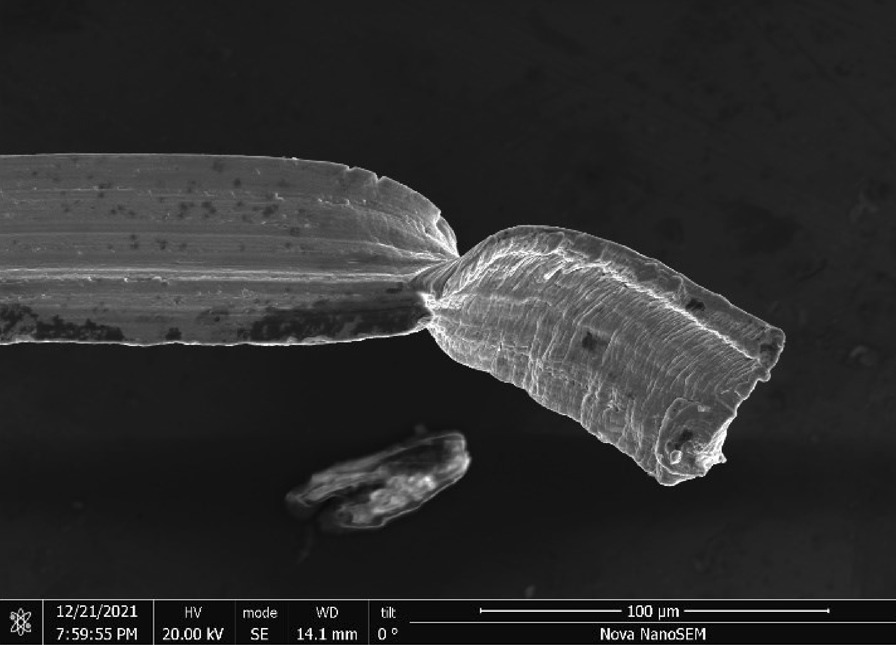
SEM-SEI image of *as-received* S.O.S. wire displaying a candy-wrapper end. Scale bar is 100 µm

S.O.S Pads—S.O.S pads (Fig. 29) are made from wires with square-like cross-sectional area of about 35 μm × 35 μm in the range of the finest human hair (Fig. 30). Cracks are prevalent along the wires due to its highly deformed state (Figs. 31 and 32). The composition is predominantly iron as expected for a carbon steel. No chemical analysis was done of the blue soap embedded in the pads, but according to the manufacturer’s web-site: “The soap contains rust inhibitors, preservatives, biodegradable soaps and detergents, a pH buffer, fragrance and color [5]”.

### ‘As-pressed’ condition

A reasonable amount of mesh material was taken and pressed by hand and push stick down the length of the Pyrex® straight tubes to the end of the tube and compacted against a glass plate blocking the open end of the tube forming a wad. This surface is referred to as the bottom surface. Pressing is a deformation process where the metal alloy wires deform both elastically (spring back) and plastically (permanent set). Imaging of the final as-pressed condition was done by optical microscopy. In some images, the wad is at the end of the tube, while in others the wad is pushed back 1 cm to simulate the approximate gap for the rock during heating.

**Fig. 33:**
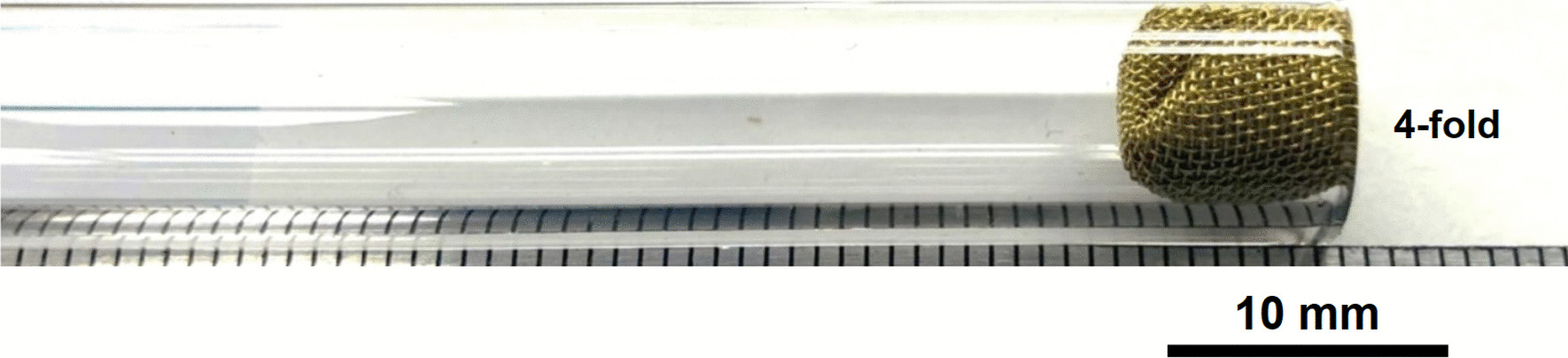
OM image of four Impact 1.0 screens folded and pressed down the stem tube

**Fig. 34:**
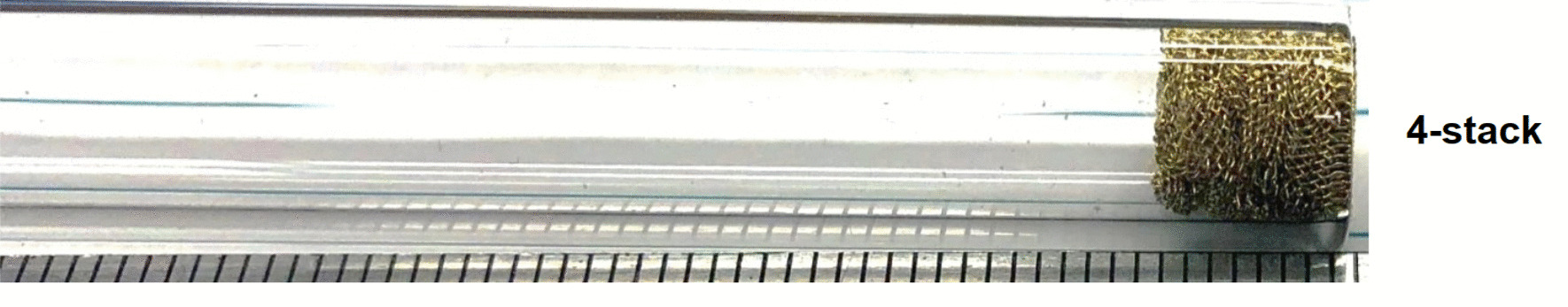
OM image of four Impact 1.0 screens stacked and pressed down the stem tube

Brass Impact 1.0—Two methods were used to press four screens into the tubes: fourfold (Fig. 33) or 4-stack (Fig. 34). In fourfold, four screens were stacked on each other and folded by hand into a small enough ball to press into the tube down to the end, flipped and then reversed back 1 cm. The fourfold screens were quite difficult to manipulate by hand, due its small mesh size, larger wire diameter, and also because of the sharp wire ends on the screen. In the 4-stack method, each screen was pushed down the tube individually and pressed onto the other forming the stack.

**Fig. 35:**
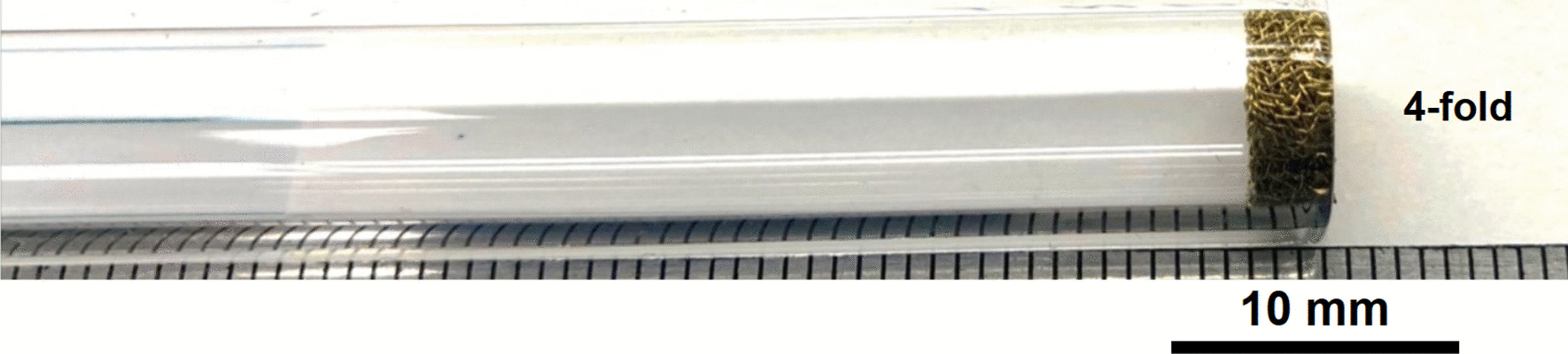
OM image of four Brass Black Packet folded and pressed down the stem tube

Brass Black Label—The fourfold method was used to create the filter (Fig. 35). The four screens were easy to manipulate and press down the tube. Compare the thickness of Fig. 35 to Fig. 34.

**Fig. 36:**
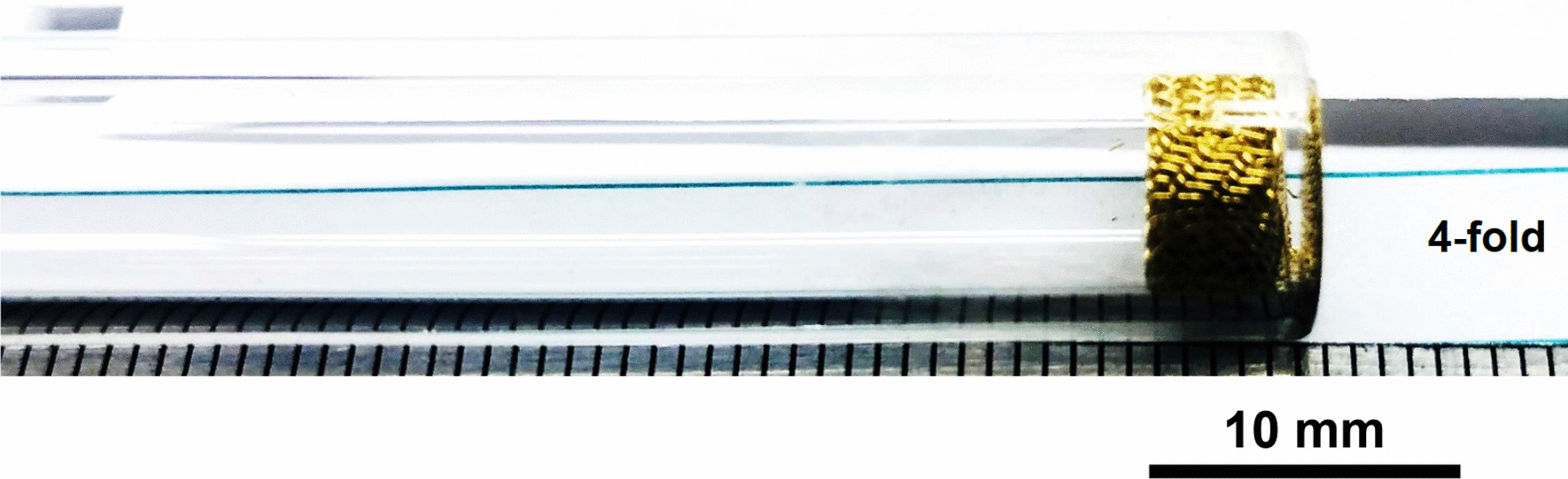
OM image of four Impact 2.0 folded and pressed down the stem tube

Brass Impact 2.0—The fourfold method was used to create the filter (Fig. 36). The four screens were easier to manipulate by hand and press down the tube then Impact 1.0.

**Fig. 37:**
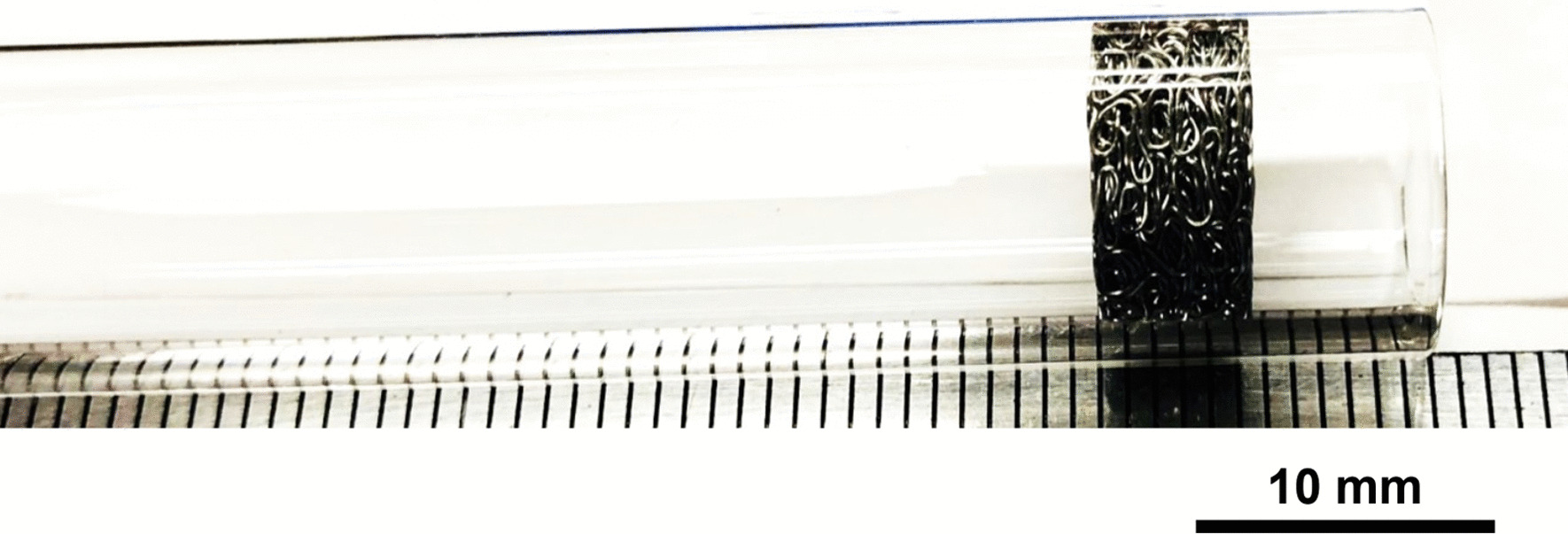
OM image of pellet screen pushed toward the end of the stem tube

Stainless Steel Pellet Screen—The stainless steel mesh was pre-shaped, had no exposed wire ends and was easy to fit in and push down the kit-provided Pyrex® tube (Fig. 37).

**Fig. 38:**
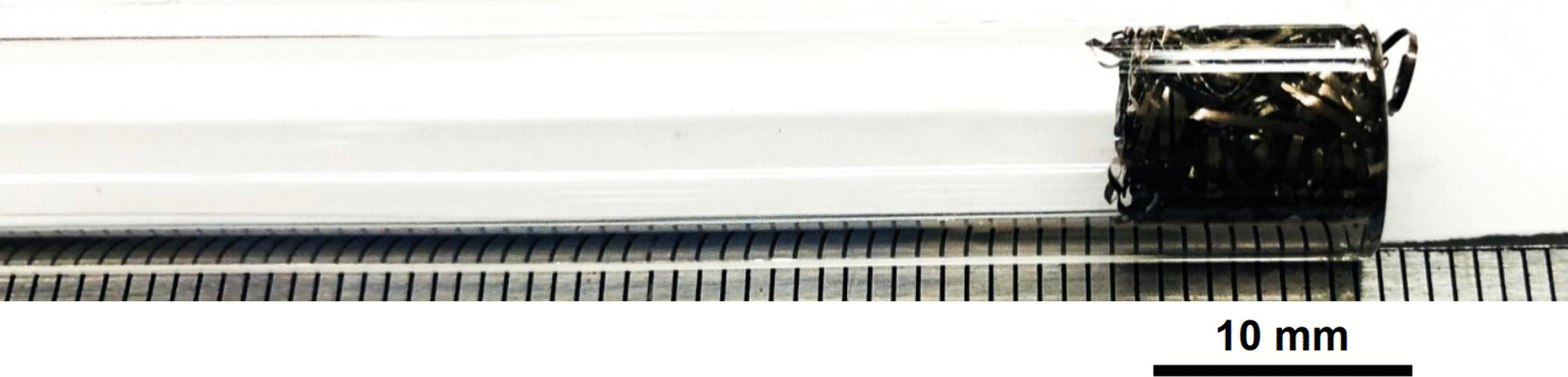
OM image of Scrubber Cleanz pressed down toward the end of the stem tube

Scrubber CleanZ—A portion of the CleanZ scrubber was cut with scissors, and shards of wire separated and fell from the wad during cutting. The material was heated with the butane lighter for 20 s to burn away any residue. Once cooled, the wad was pressed down the tube (Fig. 38). The coiled ribbon shape and stiffness of the wires made them difficult to compress down into the tube leaving considerable open gaps in the filter, and free wires dangled on both free-ends of the wad.

**Fig. 39:**
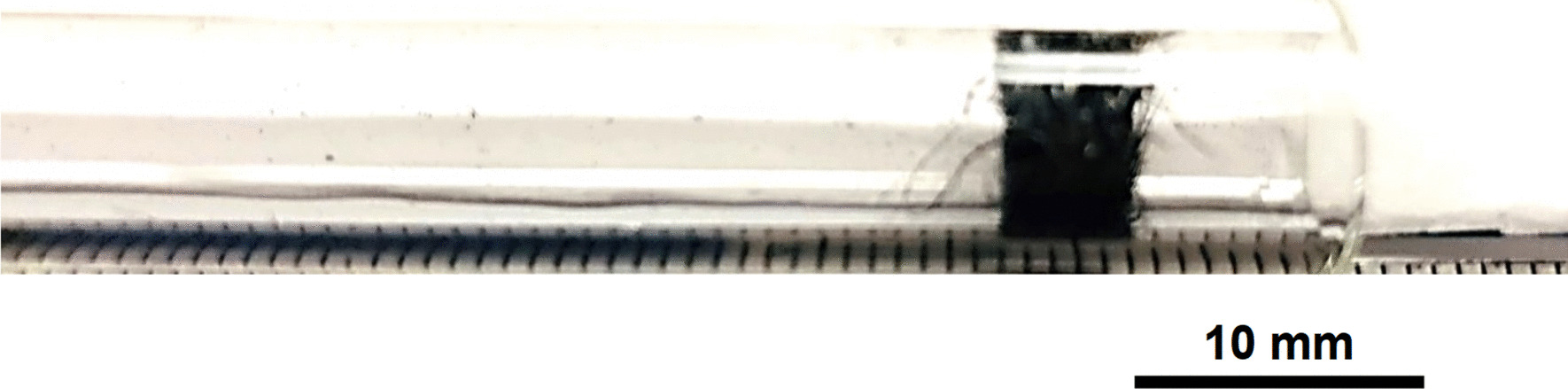
OM image of Bull Dog steel wool *as-pressed* to end of tube and back

Bull Dog—A portion of the Bull Dog wool was cut with scissors. Shards of wire fell out during the cutting. The material was exposed to the butane lighter flame for 20 s during which some of the smaller wires ignited, burned and melted. After heating and cooling, the wool was easily pressed down the tube (Fig. 39). The tube had considerable debris (small pieces of wire) visible as dark specks coating the inside of the tube after the pressing.

**Fig. 40:**
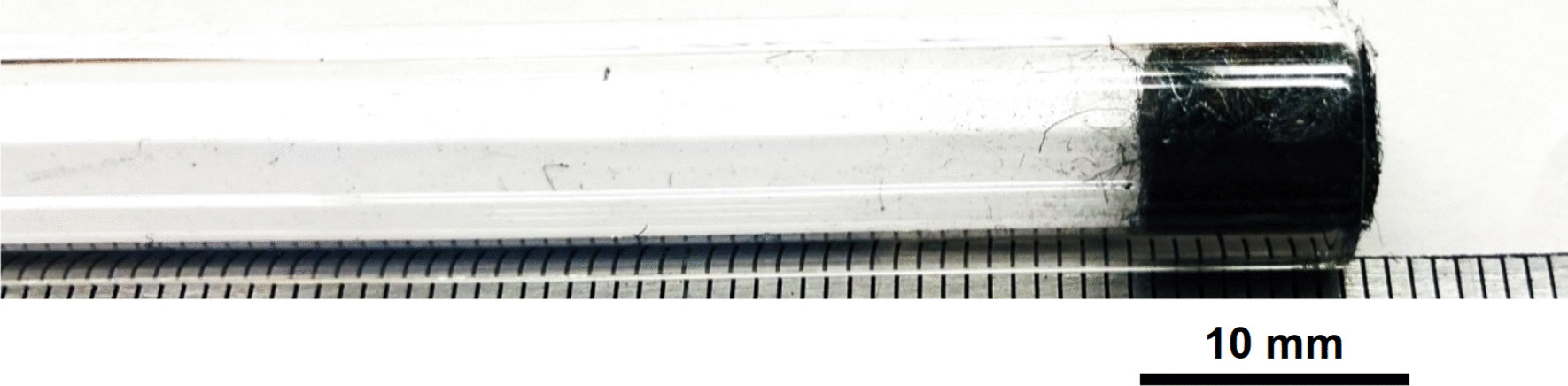
OM image of Rhodes American steel wool *as-pressed* to end of tube

Rhodes—A reasonable amount of Rhodes wool was ripped by hand from the larger amount (Fig. 25) and heated for 20 s using the butane lighter. The wool ignited and burnt to a larger degree than Bull Dog, likely due to the smaller size and larger number of free wire ends. The wad was easily pressed down into the tube with debris observed coating the tube interior (Fig. 40).

**Fig. 41:**
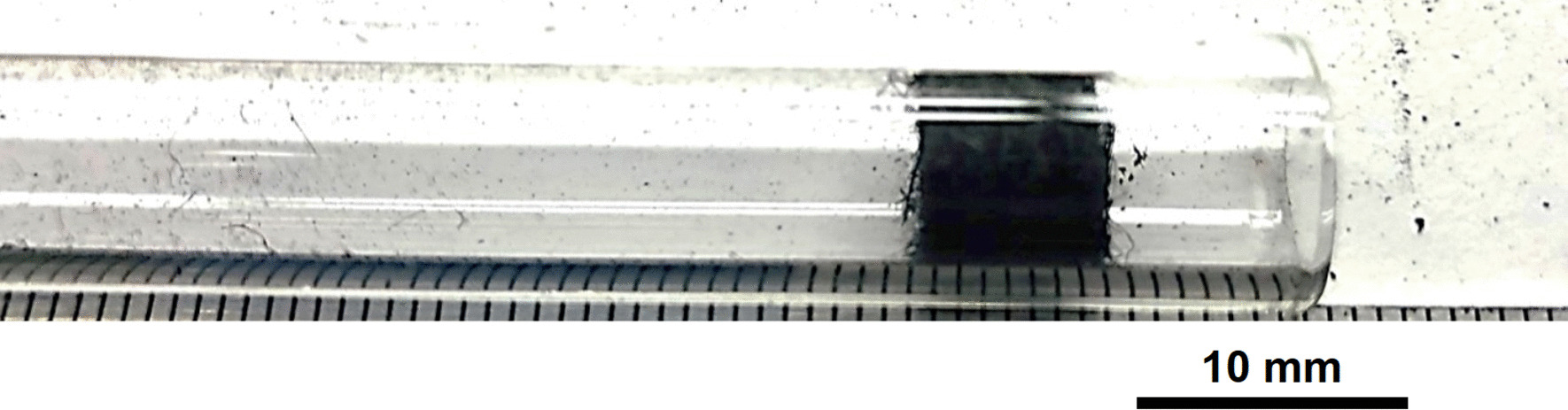
OM image of S.O.S. steel wool *as-pressed* to end of tube and back

S.O.S Pads—A portion of the S.O.S material was cut from the pad. During heating by the butane lighter, the wad caught on fire. After the wad stopped burning, the wad was easily compressed into the straight tube (Fig. 41). The tube had very fine debris coating the inside of the tube.

### ‘As-heated’ condition

OHRDP provided researchers with a demonstration video prepared by drug harm reduction workers and users, on the method of packing and duration of heating which was used for replication purposes for this research. The filter end of the pipes was heated for 20 s to simulate the time to vapourize a drug. After heating, the filter material was pushed out of the pipe and characterized by OM and SEM. All compressed wads were cleaned ultrasonically in an ethanol bath and dried before SEM imaging.

**Fig. 42:**
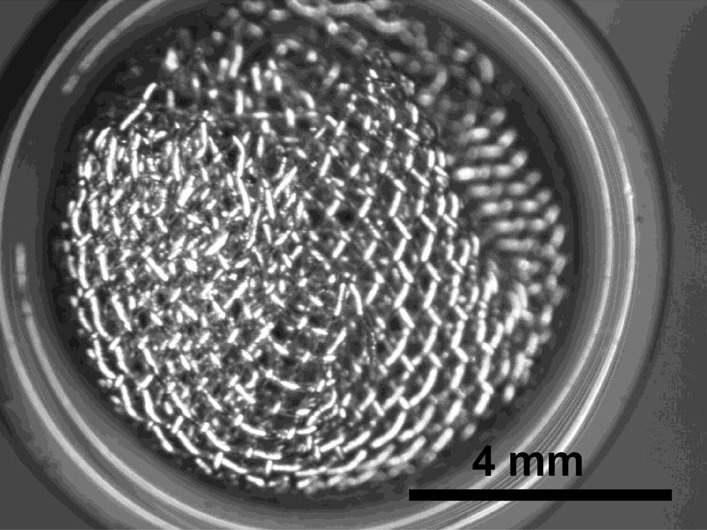
OM image of *as-heated* Impact 1.0 wad at tube end

**Fig. 43:**
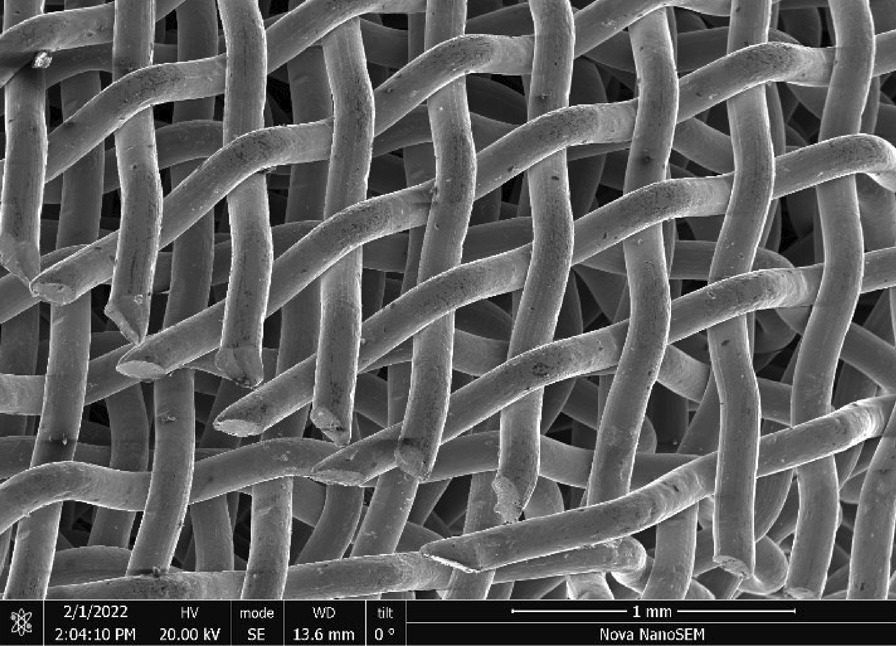
SEM-SEI image of *as-heated* Impact 1.0 wires. Scale bar is 1 mm

**Fig. 44:**
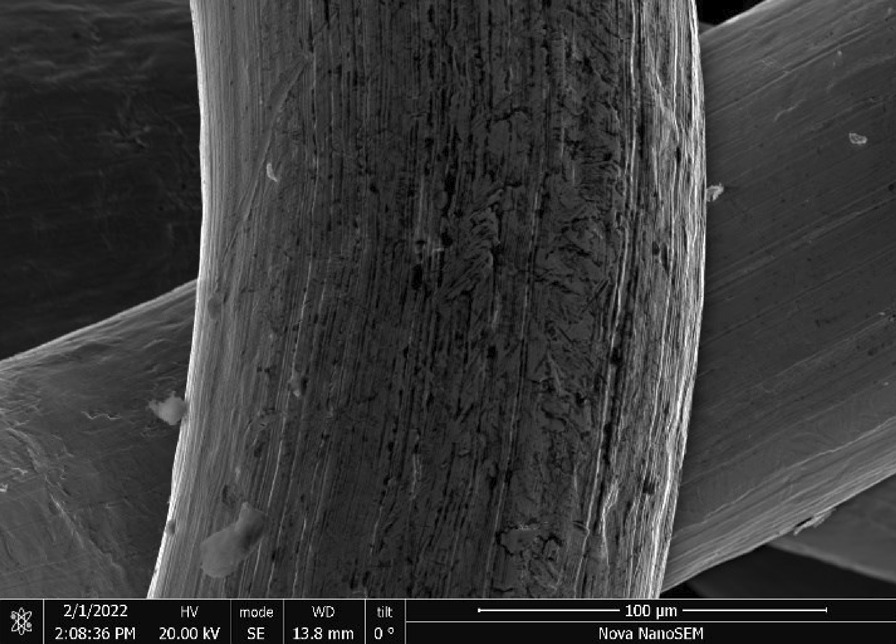
SEM-SEI image of *as-heated* Impact 1.0 wire surface. Scale bar is 100 µm

Brass Impact 1.0—Fig. 42 shows the open end of the compressed screens. The Impact 1.0 wires are noticeably bent but remain intact (Fig. 43) and clean (Fig. 44).

**Fig. 45:**
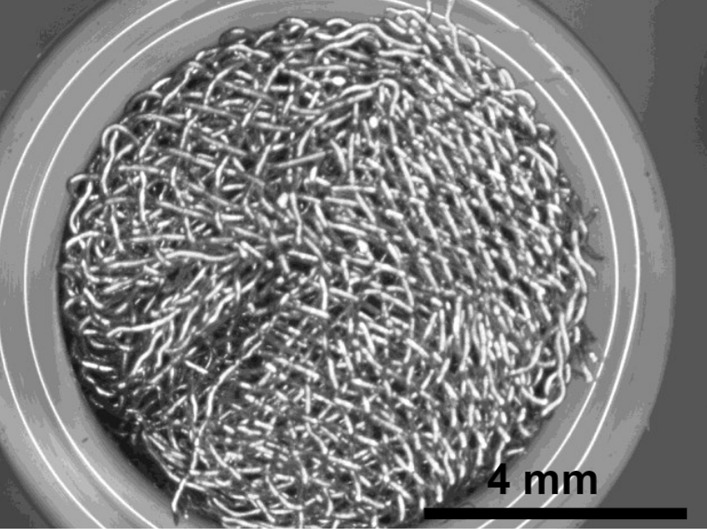
OM image of *as-heated* Brass Black Packet screen wad at tube end

**Fig. 46:**
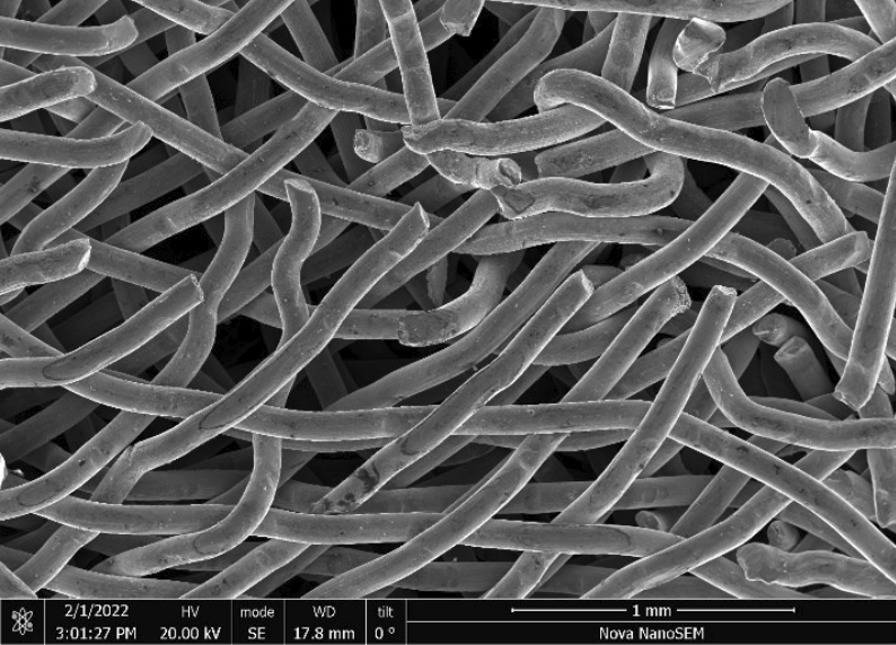
SEM-SEI of *as-heated* Brass Black Packet showing numerous fractured ends. Scale bar is 1 mm

**Fig. 47:**
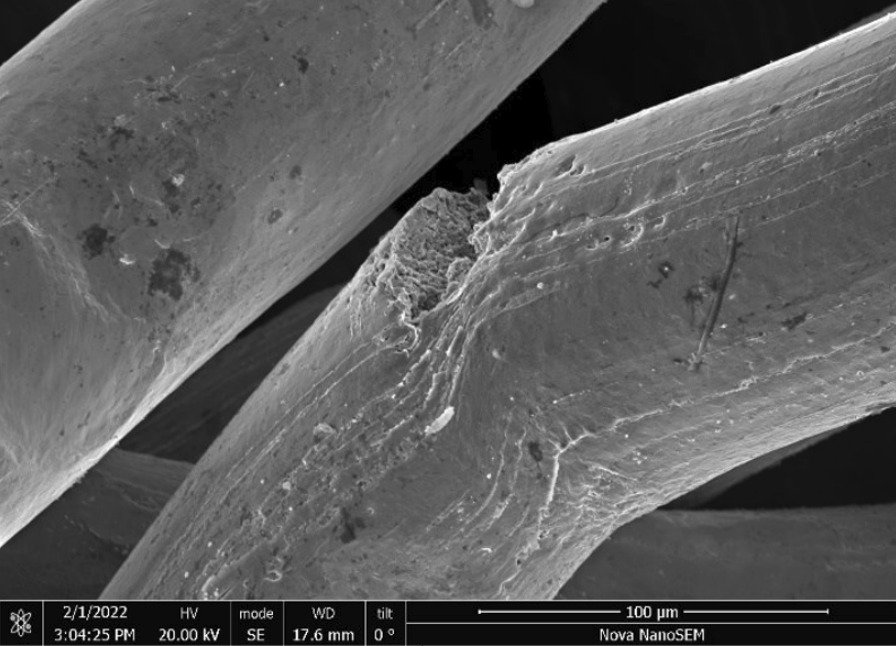
SEM-SEI of *as-heated* Brass Black Packet surface with a crack. Scale bar is 100 µm

Brass Black Label—Fig. 45 shows the open end of the compressed brass screen. There are many more kinks and breaks in the wires (Fig. 46), likely because the Black Packet brass wires were easier to deform to the breaking strain by pressing, but there is also the possibility the wires were already in a highly strained condition that limited the breaking strain. The wire surfaces appear less clean than Impact 1.0 (Fig. 47).

**Fig. 48:**
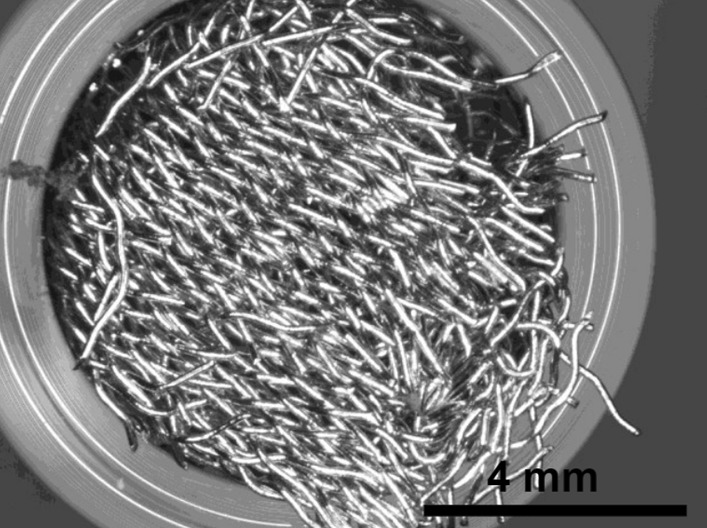
OM image of *as-heated* Impact 2.0 wad at tube end

**Fig. 49:**
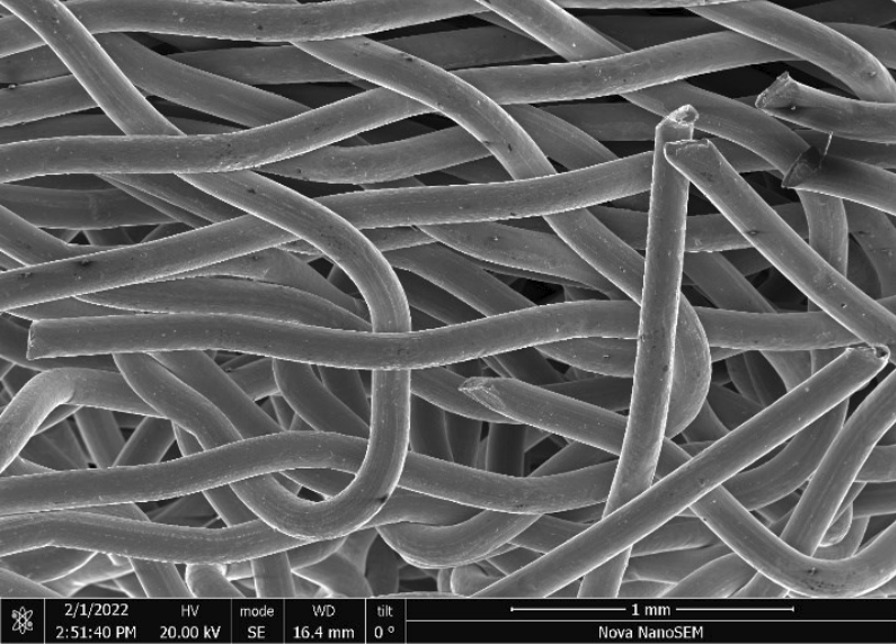
SEM-SEI image of *as-heated* Impact 2.0 wires. Scale bar is 1 mm

**Fig. 50:**
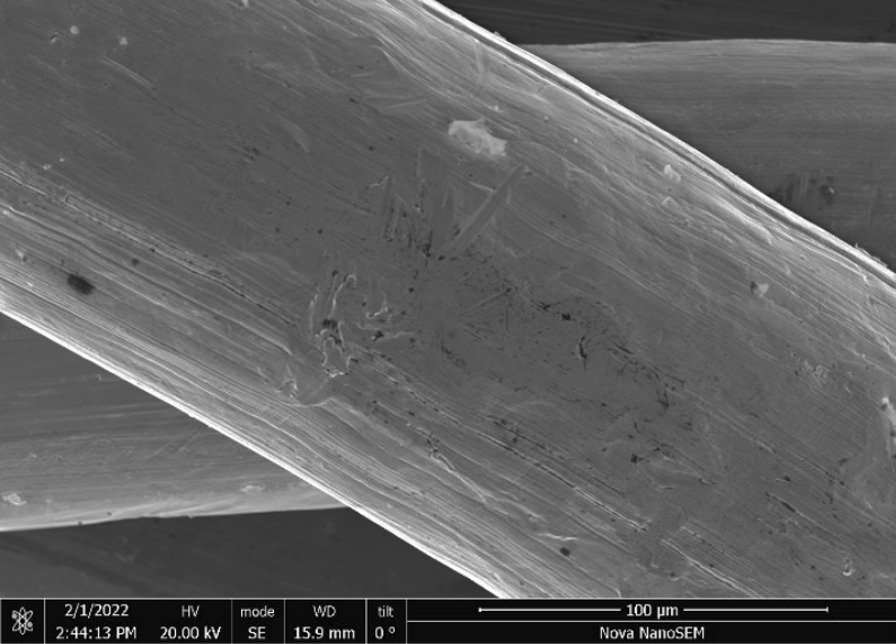
SEM-SEI image of *as-heated* Impact 2.0 wire surface. Scale bar is 100 µm

Brass Impact 2.0—Fig. 48 shows the end view of the pressed Impact 2.0 in the tube with the free-ends of the screens unravelling and spilling out of the tube. The Impact 2.0 wires were easily deformed and did not fracture after pressing (Fig. 49) and remained relatively clean after heating (Fig. 50).

**Fig. 51:**
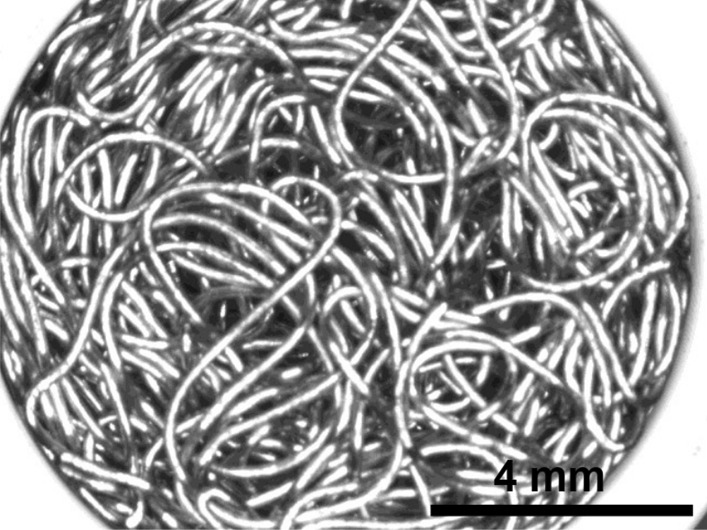
OM image of *as-heated* pellet screen at tube end

**Fig. 52:**
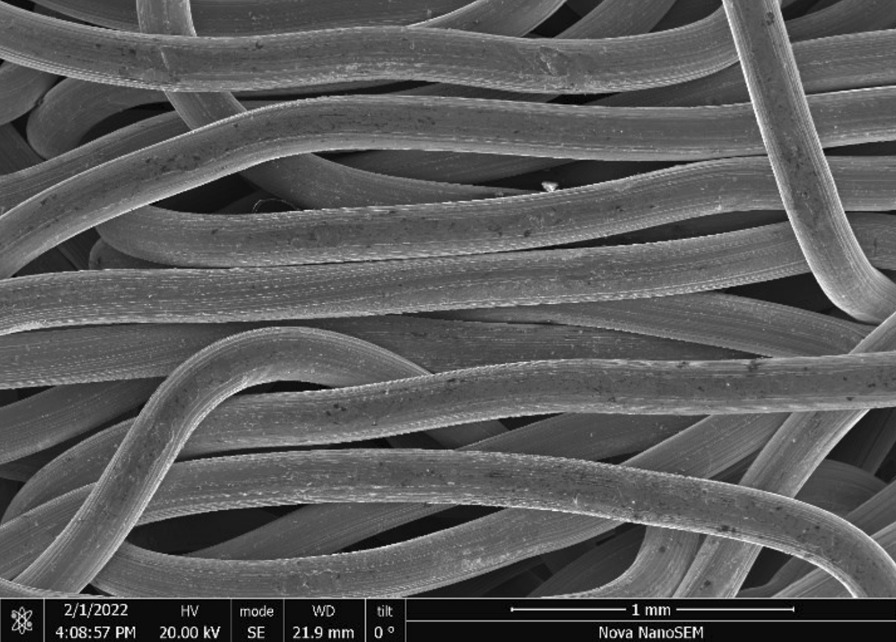
SEM-SEI image of *as-heated* pellet screen wire. Scale bar is 1 mm

**Fig. 53:**
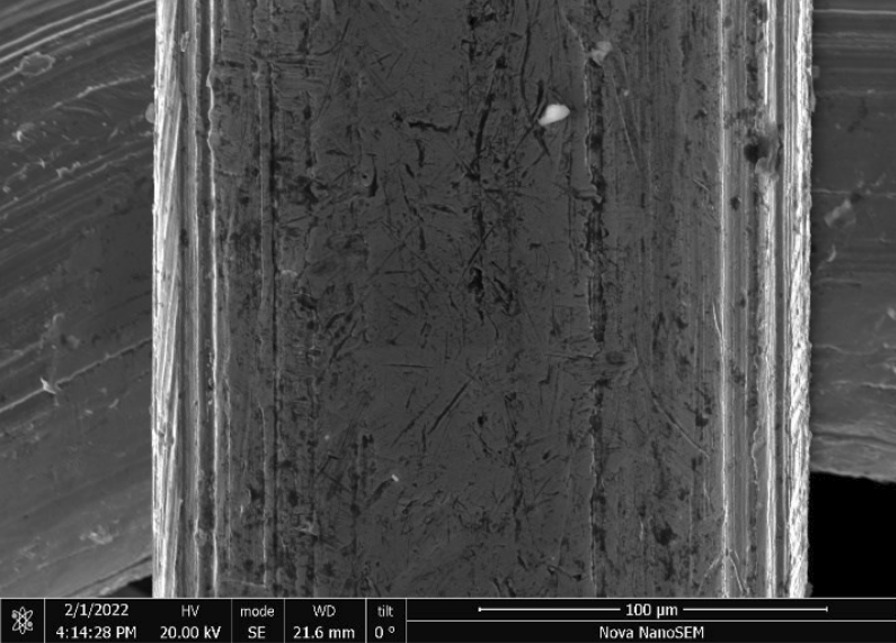
SEM-SEI image of *as-heated* pellet screen wire surface. Scale bar is 100 µm

Stainless Steel Pellet Screen—Fig. 51 shows the pellet screen at the end of the tube. The larger diameter tube size did not fit into the imaging filed leading to the cropping effect. The stainless steel pellet screen was not deformed plastically during the pressing (Fig. 52) and remained clean after the heating (Fig. 53) compared to Fig. 16.

**Fig. 54:**
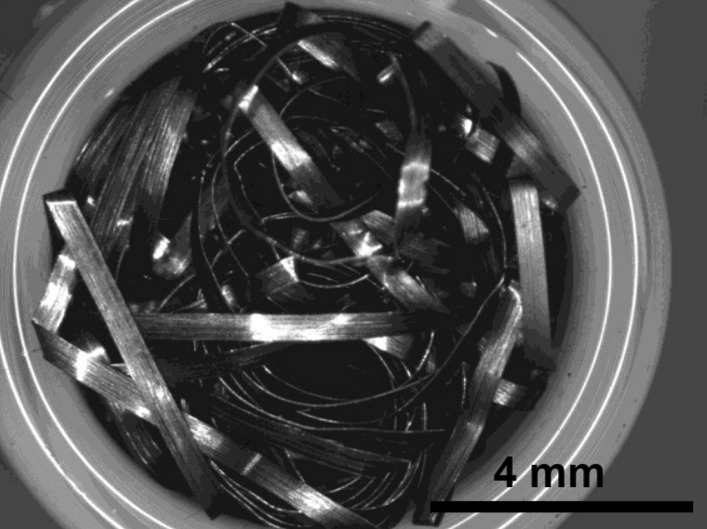
OM image of *as-heated* Scrubber CleanZ wad at tube end

**Fig. 55:**
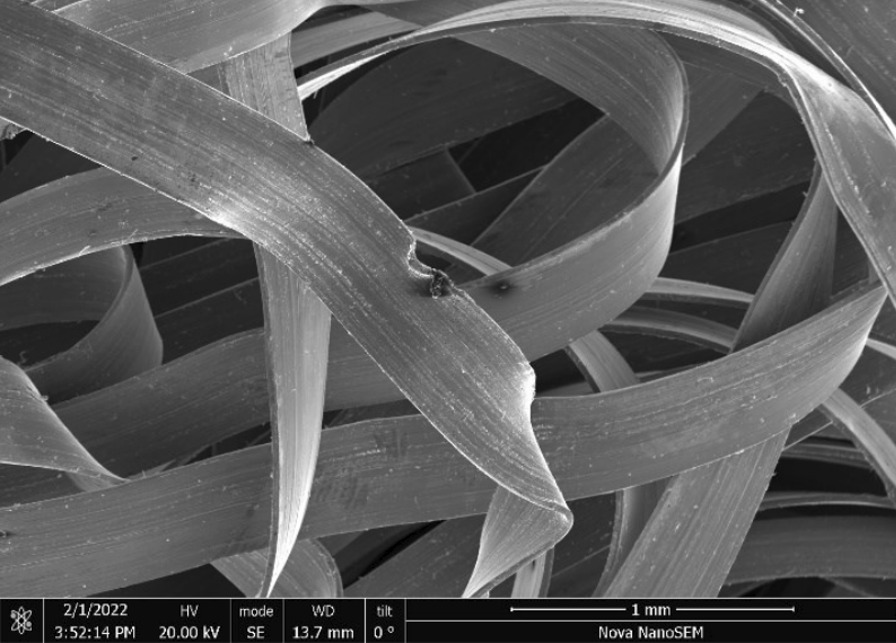
SEM-SEI image of *as-heated* Scrubber CleanZ wire ribbons. Scale bar is 1 mm

**Fig. 56:**
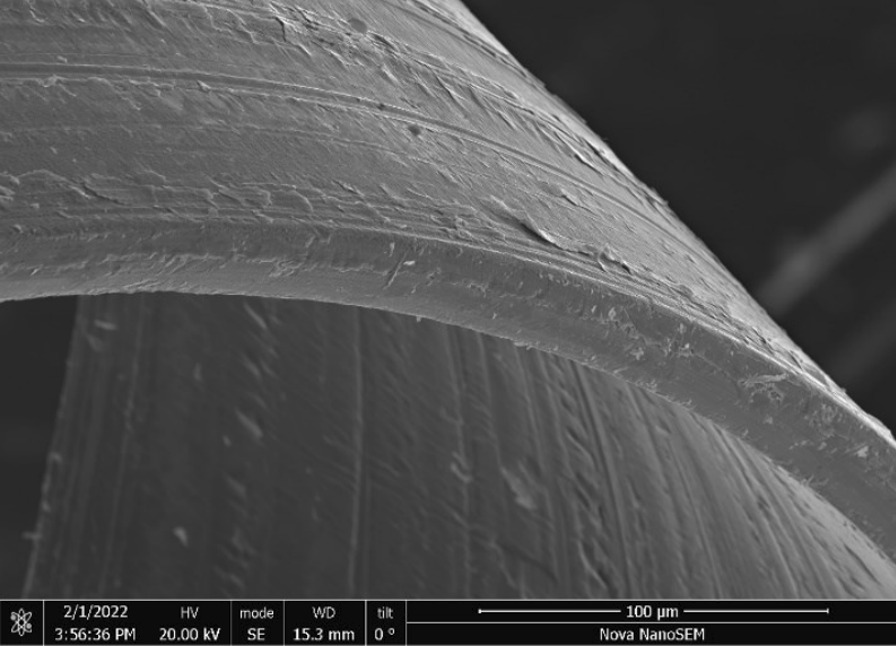
SEM-SEI image of *as-heated* Scrubber CleanZ wire ribbon surface. Scale bar is 100 µm

Scrubber CleanZ—Fig. 54 shows the *as-heated* stainless steel ribbons at the end of the tube. The wire ribbons remained intact (Fig. 55) and showed minor surface discolouring (Fig. 54) but similar roughness as *as-received* after the heating (Fig. 56).

**Fig. 57:**
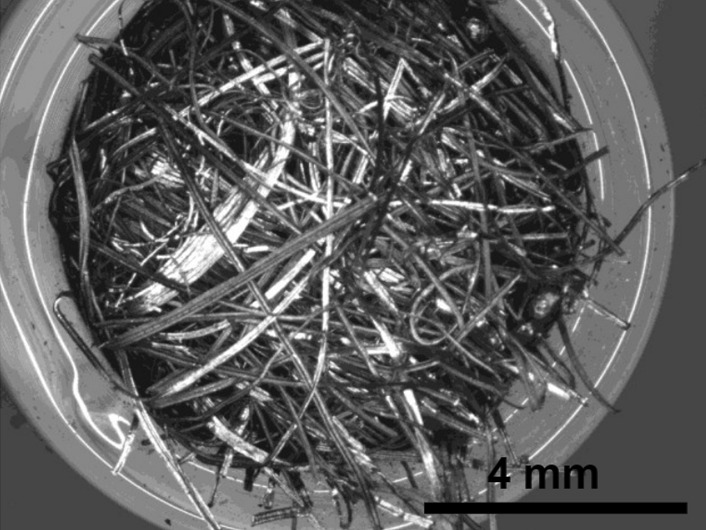
OM image of *as-heated* Bull Dog wad at tube end

**Fig. 58:**
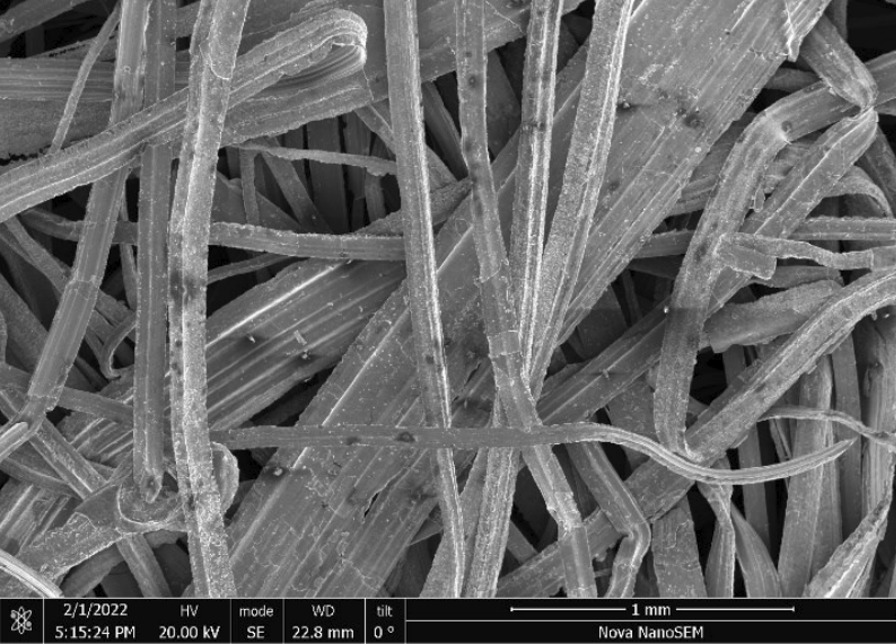
SEM-SEI image of *as-heated* Bull Dog wires. Scale bar is 1 mm

**Fig. 59:**
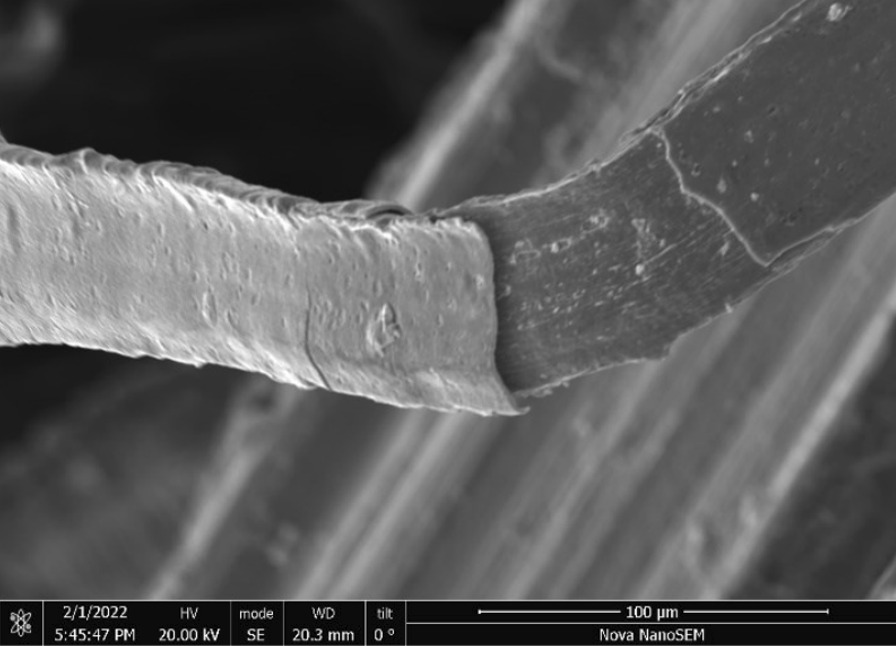
SEM-SEI image of *as-heated* Bull Dog wire surface. Scale bar is 100 µm

Bull Dog—Fig. 57 shows the wire wad spilling out of the end of the tube after pressing. Melting was detected in some wires (Fig. 58), and most of the wires were coated with a non-conducting oxide that was brittle showing cracking (Fig. 59).

**Fig. 60:**
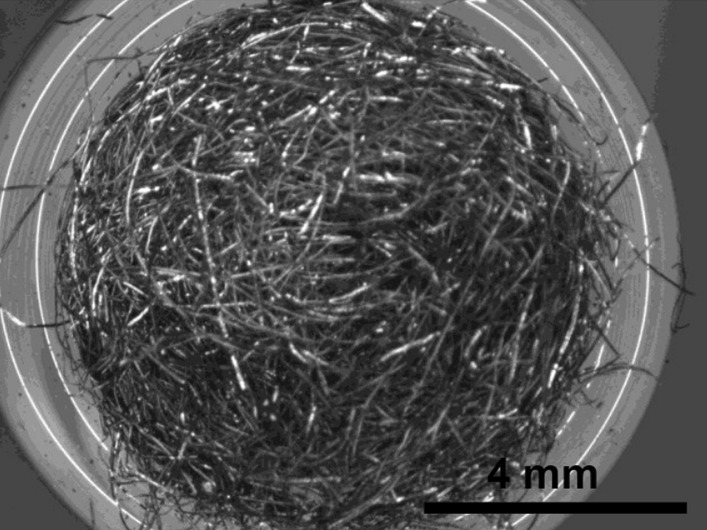
OM image of *as-heated* Rhodes American wad at tube end

**Fig. 61:**
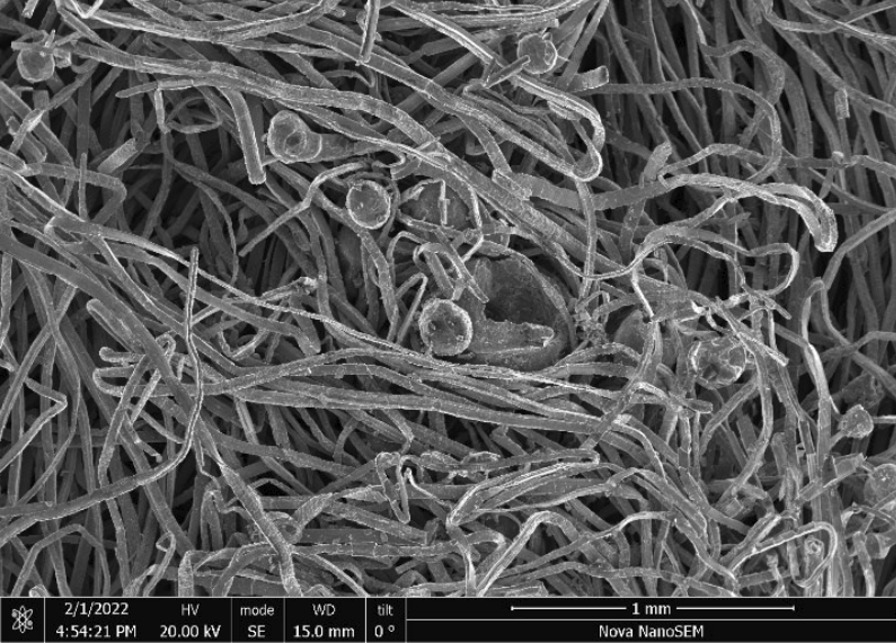
SEM-SEI image of *as-heated* Rhodes American steel wires. Scale bar is 1 mm

**Fig. 62:**
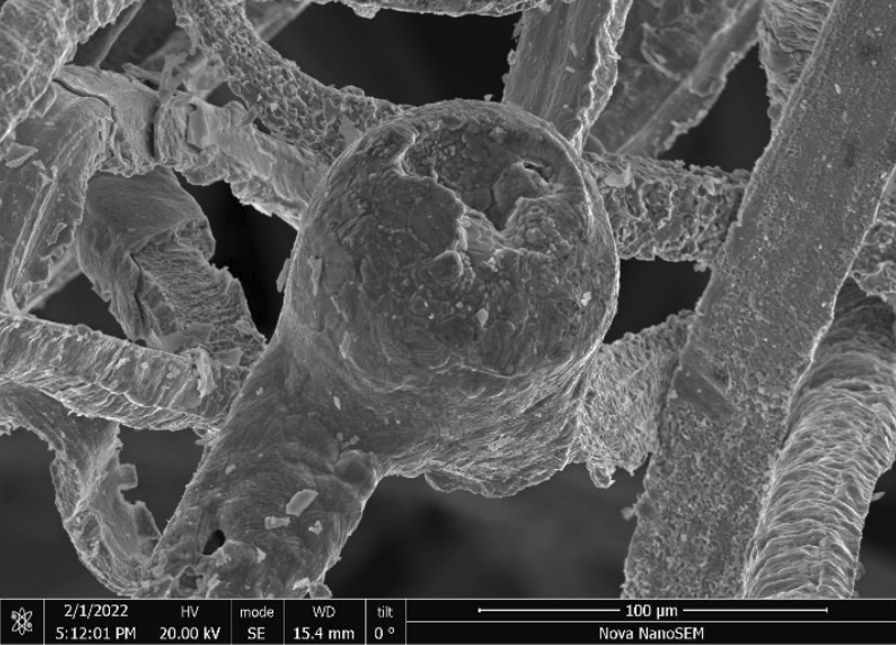
SEM-SEI image of *as-heated* Rhodes American wire surfaces. Scale bar is 100 µm

Rhodes American—Fig. 60 shows the Rhodes steel wool at the end of the tube after pressing and heating. Spheroidized melted wire tips are prevalent throughout the wool resembling balls (Fig. 61). The entire wire surfaces showed extensive oxidation (Fig. 62).

**Fig. 63:**
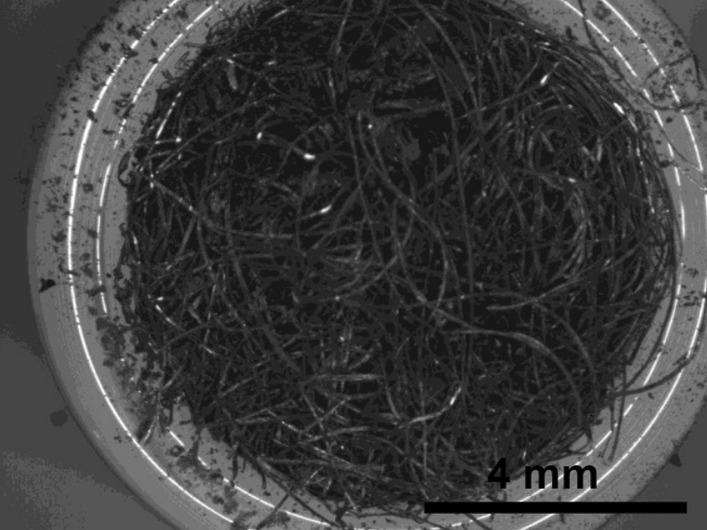
OM image of *as-heated* S.O.S. wad at tube end

**Fig. 64:**
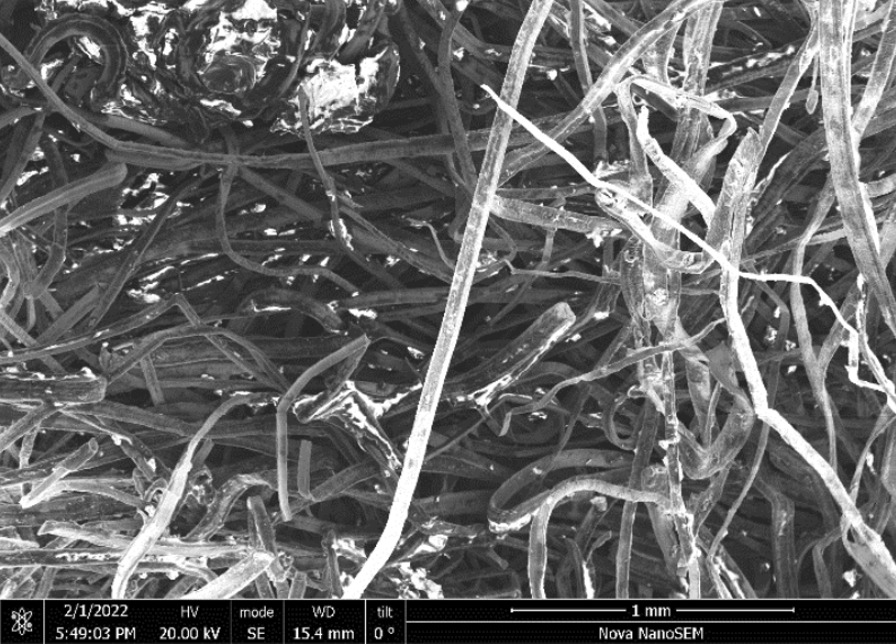
SEM-SEI image of *as-heated* S.O.S. wires. Scale bar is 1 mm

**Fig. 65:**
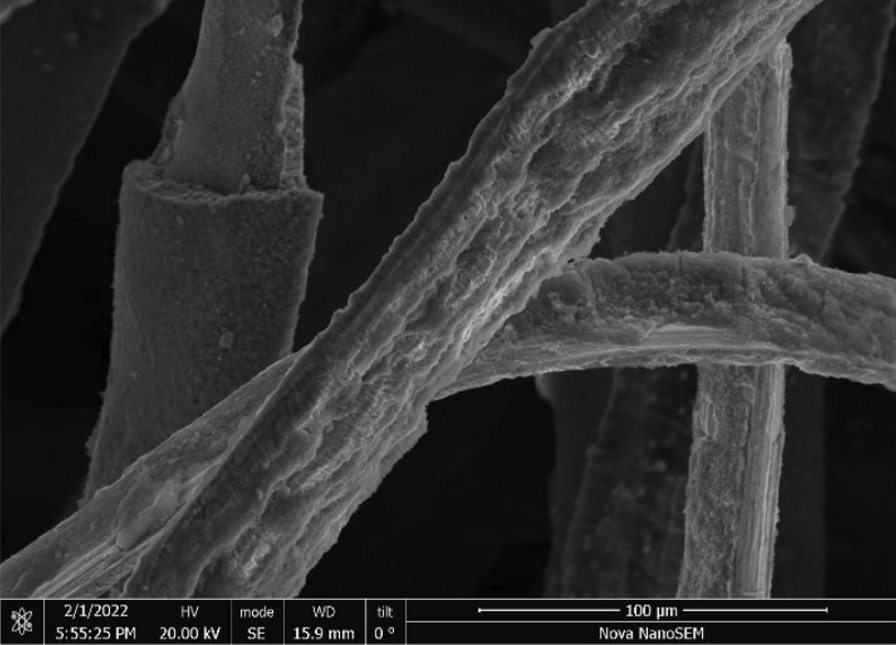
SEM-SEI image of *as-heated* S.O.S. wire surfaces showing microscopic roughness. Scale bar is 100 µm

S.O.S—Fig. 63 shows an optical image of the S.O.S wires pressed, heated and pushed to the end of the tube. The dark fine particle residue along the lip of the tube is clearly visible. The SEM image of Fig. 64 shows bent and distorted oxidized wires. The wire surfaces are non-uniform and crusty (Fig. 65).

## Discussion

The main objective of this study was to reveal the microscopic features and changes present in materials used as filter screens in straight Pyrex® stems for drug consumption before and after being manipulated and heated using a simulated technique for the preparation of drugs for smoking/inhalation. The findings from this study provide the first evidence that both the manipulation of filter material and its heating have different effects depending on the material. Consequentially, we believe that these changes in screen materials during handling and heating are likely to have an effect on the experience of smoking drugs by retaining more or less drugs inside the screen and health risks associated with inhalation/ingestion of loose fragments and smouldered crack cocaine particles. Although the experiment in this study involves a simulated method of crack cocaine preparation for smoking, the experiments were performed in the absence of the drug because the primary focus was on comparing how brass screens and steel wools behave when exposed to heat and its implications on health. What we found is that steel wools degrade faster than brass screens, creating by-products that are inhaled with the vapours. Some steel wool products were found to shrink when heated which can cause them to become loose in the stem and accidentally inhaled. Because the experiment was performed in the absence of drugs, we were unable to examine the effect of the drug vapours on the brass screens and steel wools and its impact on drug delivery. We hypothesize that increase in the surface area causes by the roughness of steel wool surface would retain more drug on the surface if the condensation of the drug vapour on the filter wires on cooling appears. As more drug condensate would remain trapped on the steel wool wire surface, less drug would be available for smoking. However, because the experiment is performed in the absence of drugs, further research is needed to determine if the coating effect on wire surface occurs. To our knowledge, this is the first study to examine the drug consumption methodology’s effects, which include manipulation and positioning of the screens in the Pyrex® stem and heating, on different materials and compare the observed effects between the brass screens and other often used steel wool alternatives. This study demonstrated that brass screens and stainless steel screens remain relatively unchanged during manipulation and heating, retaining wire dimensions and cleaner surfaces compared to steel wool alternatives.

**Fig. 66:**
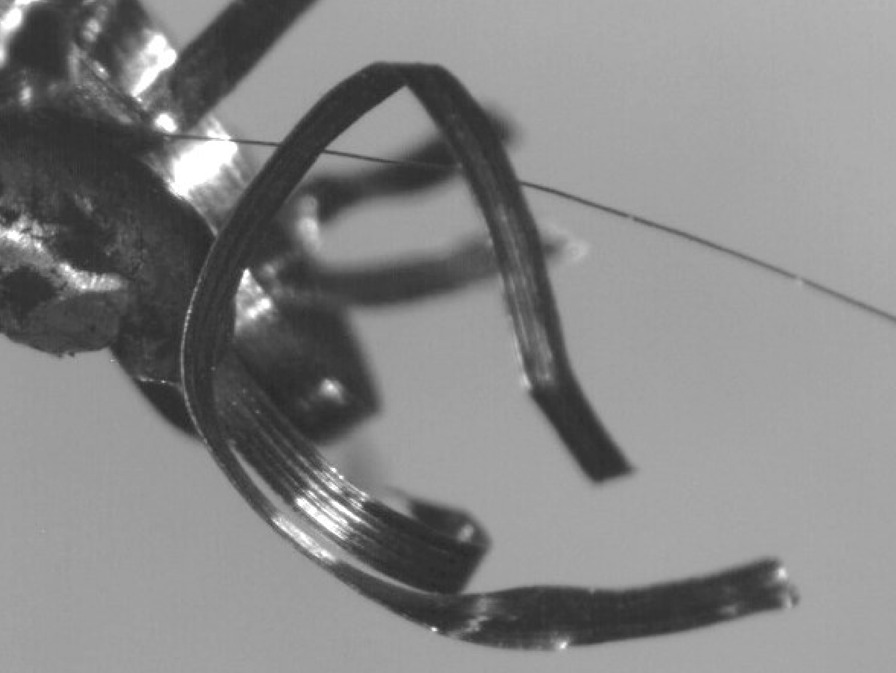
As*-heated* CleanZ fragments recovered from the tube interior by the tip of magnetized tweezers

**Fig. 67:**
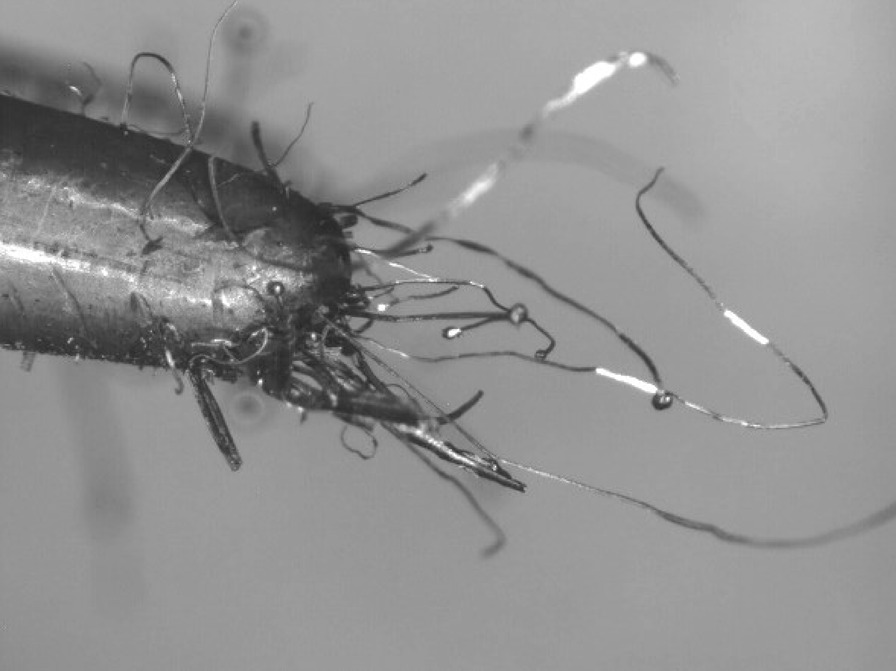
As*-heated* Bull Dog fragments recovered from the tube interior by the tip of magnetized tweezers

**Fig. 68:**
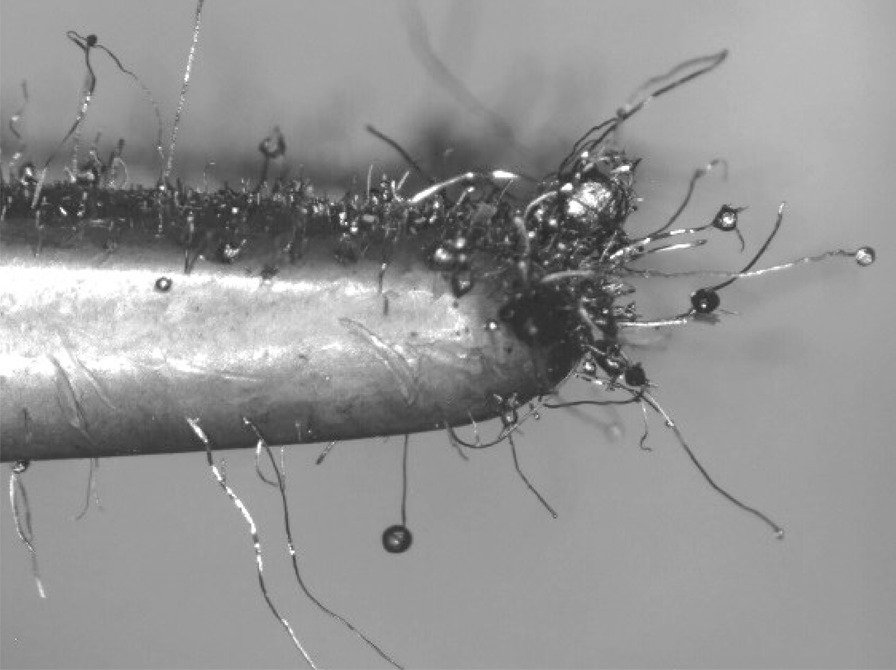
As*-heated* Rhodes American fragments recovered from the tube interior by the tip of magnetized tweezers

**Fig. 69:**
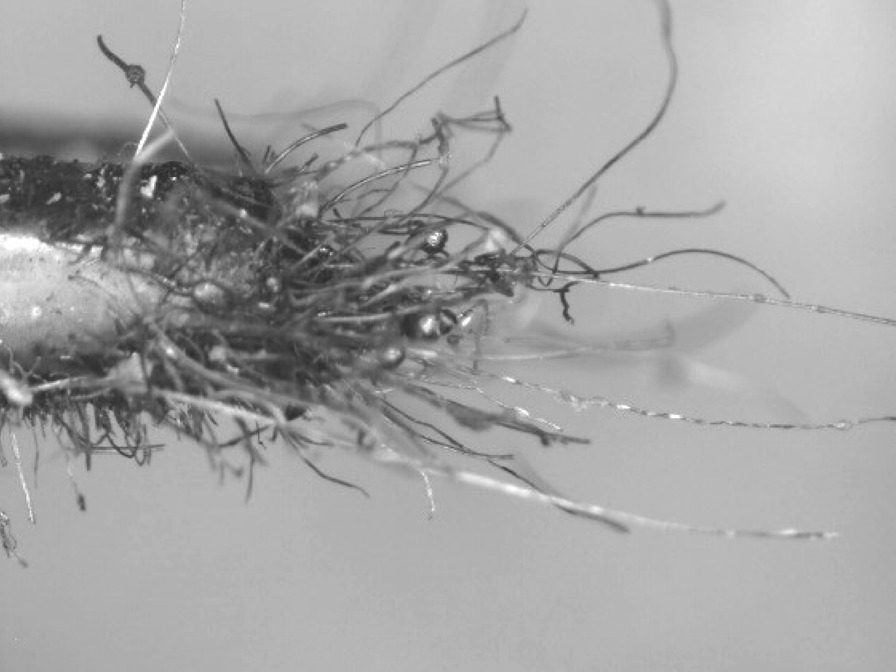
As*-heated* S.O.S. fragments recovered from the tube interior by the tip of magnetized tweezers

The wool steel wires illustrated significant structural changes like oxidation, melting and breaking into smaller segments after the same preparation. An example of the degree of smaller fibres present in *as-heated* wools can be seen in the following. Figures 66, 67, 68 and 69 show the range of wire segment pieces for the four wools picked up by the tip of magnetized tweezers. Spherical balls formed after melting and solidifying are clearly visible for Bull Dog, Rhodes and S.O.S. The wires are easily separated from the general filter wad and could be inhaled during drug consumption.

The stainless steel pellet screen was the easiest material to manipulate and press into the Pyrex® tube while the CleanZ ribbon was the most difficult. The steel wools broke into smaller wire fragments or debris during pressing into the tube, while the brass screen materials remained mostly intact after the deformation. The screen materials surfaces and wire shapes remained unchanged after 20 s of heating, but the Bull Dog, Rhodes American and S.O.S steel wool materials ignited and burned during heating leaving oxidized surfaces and brittle wires with resolidified ends that could easily tear off and be inhaled/ingested when smoking/inhaling drugs. The residues from steel wool screens remain on the inside wall of the Pyrex® stem and are likely be scraped off in the process of recovering drug residues and could be then inhaled when drug residues are smoked.

In considering how a material is used as a filter in a straight pipe stem for drug intake, three design factors should be considered. Firstly, how easy is it to manipulate the material into the tube to create the best filter to optimize the drug consumption experience? Secondly, how stable is the filter material during heating and drug use? Thirdly, what is the best filter pathway for a person to draw vapour and keep the rock fixed until it is fully consumed? Based upon the observations made, summaries of the best and worst materials can be made for the first two categories. The filter pathway and ultimately cost are outside the scope of this study.

How easy is it to manipulate the material into the tube to create the best filter to optimize the drug consumption experience? The stainless steel pellet screen was the easiest to manipulate, because it came pre-formed to fit into the supplied Pyrex® tube. In contrast, Impact 1.0 was the most difficult to manipulate into shape due to its smaller aperture size, larger wire diameter and sharp edges around the screen periphery. The exposed sharp wires on the peripheries of the brass screens can make handling the screens difficult if someone has sensitive fingertips; the stiffer Impact 1.0 screen was the most difficult to manipulate of the three brass screens. It was found that folding and pushing Impact 1.0 screens serially into the tube were easier and faster than trying to fold a stack of screens and push them down a tube. The ease of manipulation is connected to the mechanical properties of the metal wires used in the screens and the geometry of the screens. Steel is elastically stiffer and stronger than brass, but the small diameter of the wool wires makes them very easy to deform and push down into the tubes to make the filter wad. The exception is the CleanZ ribbons, which are larger than the wool wires, highly strained and difficult to shape by hand. The ease of manipulation feature along with accessibility may be factors that unfortunately make steel wools attractive for use in straight stem pipe drug consumption. A secondary effect of the wire stiffness and strength is the greater tendency of harder materials like the steels to scratch the Pyrex® stems thus affecting tube integrity.

How stable is the filter material during heating and drug use? The stability of the filter material refers to the wire shape and chemical composition. All *as-received* steel wools are characterized by a range of wire diameters and discontinuous segment lengths from their manufacturing process. Cutting a suitable amount of wool to insert into the tube creates wire debris that becomes part of the filter material. In contrast, the brass screens consist of a fixed number of wires with lengths that remain mostly intact during manipulation except for the Brass Black Packet screen ones, which were observed to fracture. Melting and burning during heating also lead to geometrical changes. Melting and re-solidifying with a new shape change the geometry. Burning leads to the reduction of wire length. Both effects were observed in the three steel wools: Bull Dog, Rhodes and S.O.S. In contrast, the brass screens were made from lower melting temperature alloys than steels, but their larger diameter wires and higher thermal conductivity make them better at dissipating heat and less likely to ignite.

Chemical composition stability refers to the ability of the material to not change composition during heating or chemical reaction with the vapour. The qualitative observations indicate that the finer steel wool wires readily ignite in air, burning and melting and leaving an oxidized and discoloured surface. Bulldog was the most flammable and degradable during heating, while the brass and stainless screen wires did not show noticeable surface changes by SEM.

In the final category, the S.O.S material generated the most surface ‘crud’, but Rhodes and Bull Dog had a significant number of short wires generated by the preparation. In contrast, the brass and stainless screens remained clean. Free wire ends are undesirable, because they are sharp to the touch, and wires that separate can become part of the intake, and wire ends can rapidly heat up, melt and burn. The stainless steel pellet screen had the largest diameter wire that appears as a single wire.

Except for the stainless steel pellet screen, the other filter materials were developed for other applications in mind and are used ad hoc for drug consumption. It is highly recommended that filter materials be specifically designed for safer drug use by clearly defining the function of the filter and considering feedback from people who smoke drugs on its use.

### Limitations

A limitation of this study is that we were not able to investigate the effect of vapourizing an actual drug on the filter materials. Further research is needed to examine the chemical reaction of the specific drug vapour on the metal alloy screen during heating which should be investigated to confirm the stability of the filter materials and its effect on the drug. Another limitation of the study is an unknown effect in the presence of the drug vapour which is the possible condensation of the drug vapour on the filter wires on cooling. We hypothesize that, assuming constant wettability for metal surfaces, the amount of drug lost by coating the wires increases as the wire surface area to volume increases like for the steel wools with significant surface roughness. If this coating effect does occur, more of the drug would remain trapped in the screen and the intensity of drug effect would be decreased for the wools compared to the screen filter materials. In addition to this effect, once the crack cocaine has been smoked, the push stick is used to push the filter up the tube in order to partially recover the residual crack cocaine vapour condensation that has hardened on the inside wall of the Pyrex® stem as the pipe cools. We hypothesize that drug condensate on the interior wire surfaces would not be recovered by this scraping action reducing the amount of drug that would be available to the user.

## Conclusions

The high prevalence among people who smoke drugs of using steel wools as screens/filters emphasizes the need for understanding the mechanisms of how these materials cause harm and how they compare to safer and recommended alternatives such as brass screens. Based on the findings of this study, brass screens are not only considered a safer alternative to steel wools but are likely to retain less drugs on its surface ensuring that the loss of drug when smoking is less. Furthermore, differences in the geometry of brass screens tested clearly show for the first time that manipulation and insertion into the straight stem is affected by the mesh geometry. Therefore, the user experience of manipulating and inserting screens into a stem tube could be improved by screen design.

Finally, since safety and accessibility might not be the only factors that would lead to higher uptake of brass screens, but individual’s preferences and habits have an important role in the continuation of using steel wools (Boyd et al. [27]), it is important that the provision of brass screens is coupled with educational interventions and targeted messaging. A continuous exploration of learning opportunities, barriers to change and feedback from clients regarding the use of screens are important in shaping the educational interventions. In order to shift personal drug smoking practices, in addition to safer drug smoking messaging, peers and outreach workers could consider providing demonstration to clients on how to assemble kit contents (e.g. fold and insert brass screens into the straight stem) and education regarding the rationale for using brass screens instead of other screen alternatives. In policy development, encouraging manufacturer product label warnings for products not intended for filtering inhalable vapours might also help. Providing people who smoke drugs with high-quality screens, targeted educational interventions and repeated messaging related to the use brass screens and their benefits are instrumental in helping to reduce the dependence on using unsafe alternatives and the unintended negative health consequences associated with their use.

## Figures and Tables

**Fig. 70:**
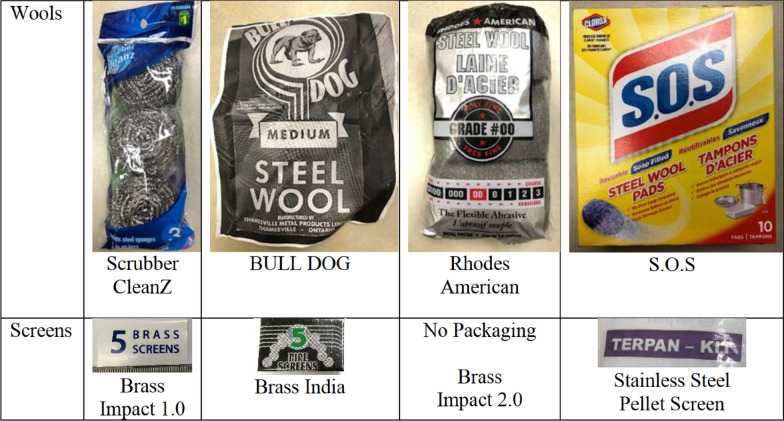
Filter material packaging

**Fig. 71:**
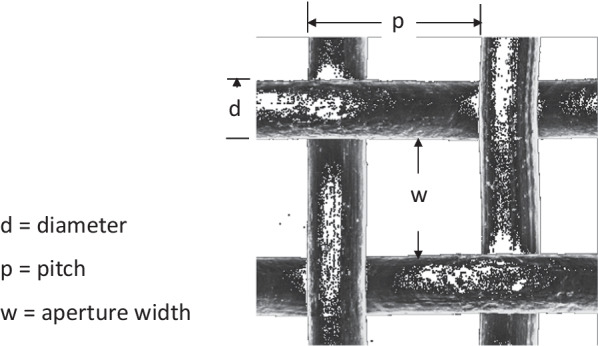
Square screen geometry

## Data Availability

Not applicable.

## Appendices

### Appendix A

See Figs. 70

### Appendix B

See Figs. 71

## Abbreviations

Brass: Copper and zinc containing metal alloy
Ferritic stainless steel: A magnetic form of stainless steel
Melting temperature: Temperature at which a solid starts to melt
Mesh: Number of openings (apertures) per inch in a screen
Micrometer: μM = 0.001 mm
OHRDP: Ontario Harm Reduction Distribution Program
OM: Optical microscopy
SEI: Secondary electron image
SEM: Scanning electron microscopy
Stainless steel: Chromium and nickel containing iron alloy
Steel: Iron alloy containing some carbon
